# Supplementary material for: Size-control in the synthesis of oxo-bridged phosphazane macrocycles via a modular addition approach
Source: Commun Chem. 2021 Feb 22;4:21. doi: 10.1038/s42004-021-00455-9 (PMC9814222; doi:10.1038/s42004-021-00455-9)
Supplement: Supplementary file 1 — Supplementary Information [file 42004_2021_455_MOESM1_ESM.pdf]

Size-control in the synthesis of oxo-bridged phosphazane macrocycles via a modular addition approach

Xiaoyan Shi,[†,a,b] Felix León,[†,a] How Chee Ong,[a] Rakesh Ganguly,[a,c] Jesús Díaz,[d,*] and Felipe García[a,*]

[a] Division of Chemistry and Biological Chemistry. School of Physical and Mathematical Sciences. Nanyang Technological University.21 Nanyang Link 637371, Singapore (Singapore).
[b] School of Materials and Energy, Guangdong University of Technology, Guangzhou,510006, Guangdong, P. R. China.
[c] Shiv Nadar University, Gautam Buddha Nagar, India 201314
[d] Departamento de Química Orgánica e Inorgánica. Facultad de Veterinaria Universidad de Extremadura, Avda de la Universidad s/n, 10003, Spain.

*E-mail: jdal@unex.es and fgarcia@ntu.edu.sg

[†] Both authors contributed equally

1 Supplementary Methods **2**

2 Characterisation of Compounds

2.1 NRM spectra **8**

2.2 FTIR and HRMS spectra **17**

2.3 X-ray analyses **21**

3 Theoretical Studies – Supplementary Discussion **26**

4 Supplementary References **37**

1 Supplementary Methods

Compounds **1-8** were prepared under dry, O_2-free Ar atmosphere on a double manifold (argon/vacuum) line. All solvents (toluene, THF, n-hexane) were freshly distilled over appropriate drying agents (sodium/benzophenone) under nitrogen atmosphere, degassed and stored under molecular sieves. Starting material were either synthesized as described below or obtained commercially from Strem, Sigma-Aldrich, Alfa-Aesar and used without further purification, unless otherwise stated. PCl_3, Et_3N were distilled from calcium hydride before use and stored under argon and molecular sieves. Starting material $[ClP(\mu\text{-}N^tBu)]_2$ was synthesized by the reaction condensation of PCl_3 with primary amines in the presence of excess triethylamine as the Brønsted base as reported.[1] Compounds **1-8** were isolated and characterized with the aid of an Ar-filled innovative technology glove box. ^{1}H, ^{13}C and ^{31}P{H} NMR spectra were recorded on Bruker BBFO 400 MHz spectrometer in the appropriate deuterated solvent (using the solvents resonances as the internal standard for ^{1}H and ^{13}C NMR and 85% H_3PO_4 – D_2O as the external standard for ^{31}P NMR). In situ ^{31}P NMR spectroscopic studies on reaction mixtures in non-deuterated solvents were recorded using an internal d_6-acetone capillary to obtain a lock. Single crystal X-ray diffraction was carried out with Bruker X8 CCD diffractometer. Shimadzu IR Prestige-21 FTIR Spectrometer was used to record the IR data and elemental analysis data performed using Euro Vector Euro EA Elemental Analyzer (CHNS).

1.1 Synthesis of $\{P(\mu\text{-}N^tBu)\}_2(\mu\text{-}O)_2\{P(\mu\text{-}N^tBu)_2PCl\}_2$ (3) (see Supplementary Scheme S1)

Synthesis of **3**: H_2O (131 μL, 7.3 mmol) and Et_3N (1.1 mL, 7.9 mmol) in 20 mL freshly distilled THF was added dropwise slowly to a solution of $[ClP(\mu\text{-}N^tBu)]_2$ (1.0 g, 3.65 mmol) in THF (20 mL) at -78 ^{o}C. The mixture was warmed back to 0 ^{o}C and stirred for 30 min and then cooled down to -78 ^{o}C again. ^{n}BuLi in hexane (6 mL, 15 mmol) was added and the reaction allowed to warm back to room temperature for 16 h. The solution was cooled to -78 ^{o}C and

added dropwise slowly to a well-stirred concentrated solution of [ClP(μ-N^{t}Bu)]$_2$ (2.0 g, 7.3 mmol) in THF (5 mL) at -78 ^{o}C. The reaction mixture was allowed to warm back to room temperature and stirred for 4 h. ^{31}P-{^{1}H} NMR (162 MHz, *in situ* in THF, δ): ~198.4 (m), 162.5 (m), 141.4 (m). Compound **3** can be isolated by filtering in celite (hexanes), but unfortunately, it could not be crystallized. Hence, throughout our report, it was produced and utilized *in situ.*

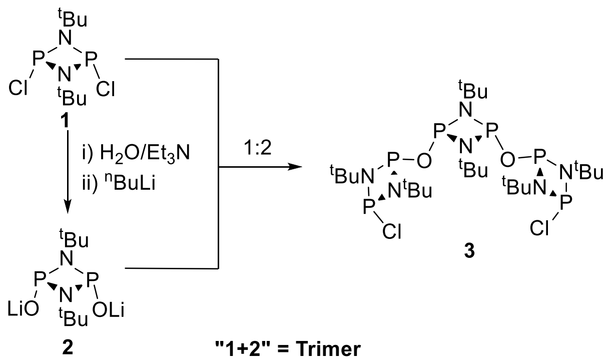

Supplementary Scheme S1

1.2 Synthesis of {P(μ-N^{t}Bu)}$_2$(μ-O)$_2$[{P(μ-N^{t}Bu)}$_2$NH^{t}Bu]$_2$ (4) (see Supplementary Scheme S2):

A solution of *tert*-butylamine (0.77 mL, 7.3 mmol) and Et$_3$N (1.1 mL, 7.9 mmol) in 20 mL freshly distilled was added slowly dropwise to the *in situ* generated THF solution of compound **3** above at -78 ^{o}C. The reaction mixture was allowed to warm back to room temperature and stirred for 4 h. The solvent was removed under vacuum, followed by extraction with toluene and filtered through a needle filter. The filtrate was concentrated in vacuo until precipitation was observed. The mixture was heated to reflux and minimum toluene was added to redissolve all the precipitate. The concentrated clear solution stored at room temperature provided high-quality crystals of **4**. Crude yield: 2.51g, 87%; Yield of the first batch of pure crystalline material: 1.61 g, 2.04 mmol (56 %). Mp: 156 °C. ^{31}P-{^{1}H} NMR (162 MHz, C$_6$D$_6$, δ): 140.1 (d, *J* = 79 Hz), 139.2 (d, *J* = 79 Hz), 119.2 (s). ^{1}H NMR (400 MHz, C$_6$D$_6$, δ): 3.38 (d, 2H, $^2J_{P\text{-}H}$ = 1.2 Hz), 1.56 (s, 18H), 1.50 (s, 36H), 1.28 (d, 18H). ^{13}C NMR (101 MHz, C$_6$D$_6$, δ): 52.4 (t, *J* = 11.6 Hz), 51.7 (d, *J* = 13.1 Hz), 33.2 (d, *J* = 9.1 Hz), 31.9 (s), 31.8 (t, *J* = 6.6 Hz). IR (Nujol, NaCl) ν (cm^{-1}): 1011 (s, P-O), 795 (s, ν(P-N)) cm^{-1}. MS (EI) m/z: 789.45 [M+H]$^+$.

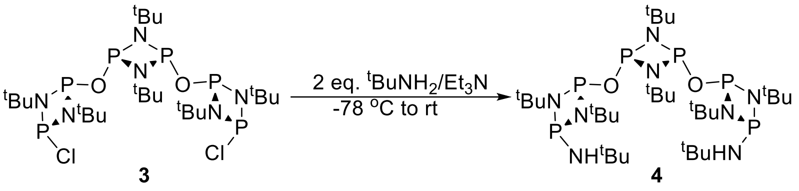

Supplementary Scheme S2

1.3 Synthesis of {P(μ-N^{t}Bu)}$_2$(μ-O)$_2${P(μ-N^{t}Bu)$_2$P(O)H}$_2$ (5) (see Supplementary Scheme S3)

H_2O (131 μL, 7.3 mmol) and Et_3N (1.1 mL, 7.9 mmol) in 20 mL freshly distilled THF was added dropwise slowly to a solution of in-situ generated **3** in THF at -78 ^{o}C. The mixture was warmed back to 0 °C and stirred for 30 min to afford compound **5**. The reaction mixture was evaporated, toluene was added (20 mL) and the suspension filtered in celite. The celite was washed with toluene (2x20 mL) and the filtrate evaporated *in vacuo* obtaining **5** as a white solid (1.8 g, 89%). High-quality crystals were obtained by dissolving the compound in toluene and storage at -25 °C overnight.^{1}H NMR (500 MHz, C_6D_6, δ): 7.55 (d, $J_{H\text{-}P}$ = 584 Hz), 1.49 (s), 1.40 (s). NMR (202 MHz, C_6D_6, δ): 139.5, 97.4, -4.8 (dd, $J_{P\text{-}H}$ = 584 Hz, $J_{P\text{-}P}$ = 14 Hz). MS (EI) m/z: 679.2901 $[M+H]^+$

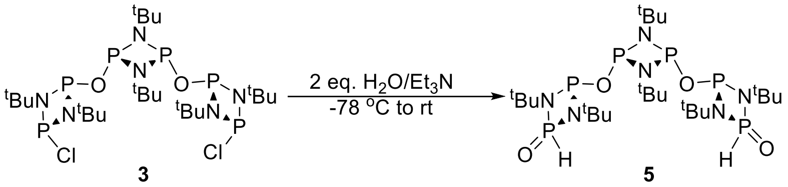

Supplementary Scheme S3

1.4 Synthesis of {P(μ-N^{t}Bu)}$_2$(μ-O)$_2${P(μ-N^{t}Bu)$_2$POM}$_2$ [M= Li (6) and K(6b)] (see Supplementary Scheme S4)

Synthesis of 6: H_2O (131 μL, 7.3 mmol) and Et_3N (1.1 mL, 7.9 mmol) in 20 mL freshly distilled THF was added dropwise slowly to a solution of *in situ* generated **3** (3.75 mmol) in THF at -78 ^{o}C. The mixture was warmed back to 0 ^{o}C and stirred for 30 min to afford compound **5**. The solution containing **5** was then cooled back down to -78 ^{o}C. ^{n}BuLi in hexane

(6 mL, 15 mmol) was added and the reaction allowed to warm back to room temperature for 16 h generating a solution of **6** in THF. Unfortunately, **6** could not be isolated for further analysis. Hence, throughout our report **6** was produced and utilized *in situ*. ^{31}P-{^1H} NMR (202 MHz, *in situ* in THF, δ): ~139.3 (dd, *J* = 84.8 Hz, *J* = 24.2 Hz), 97.7 (b), 97.4 (d, *J* = 34.3 Hz) and -3.3 ppm (d, *J* = 12.1 Hz), -3.4 ppm (d, *J* = 14.1 Hz); HRMS: m/z: 679.29 $[M+1]^+$) and **5** ($^{31}P\{^1H\}$ NMR (162 MHz, *in situ* in THF, δ): ~134.7 and 128.6 ppm; HRMS: m/z: 691.30 $[M+H]^+$

Synthesis of 6b:

In situ generated **3** in THF was added to a solution of NaHMDS (2.2 eq.) in THF at -78 ^oC dropwise. The reaction mixture was allowed to warm to room temperature and was stirred overnight. The solvent was removed under vacuum and extracted with 20 mL toluene and filtered through Celite (P3). ($^{31}P\{^1H\}$ NMR (162 MHz, *in situ* in toluene, δ): ~178.3 (d. *J* = 108 Hz), 157.4 (d, *J* = 108 Hz), 151.8 (bs)

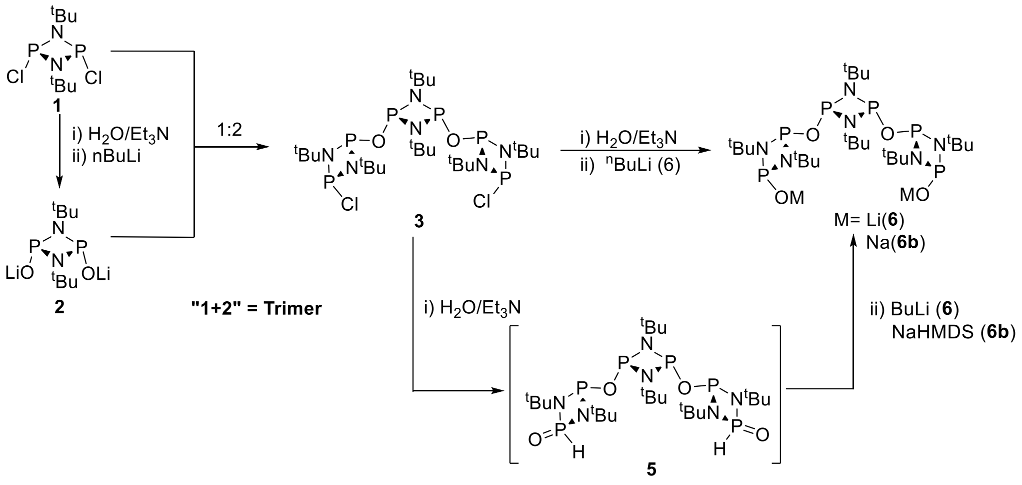

Supplementary Scheme S4

1.5 Synthesis of {(μ-O)[P(μ-N^{t}Bu)]$_2$}$_6$ (7) (see Supplementary Scheme S5)

In situ generated **3** in THF was added to a solution of *in situ* generated **6** in THF at -78 ^oC dropwise. The reaction mixture was allowed to warm to room temperature and was stirred overnight. The solvent was removed under vacuum and extracted with 20 mL *n*-hexane and

filtered through Celite (P3). The filtrate was concentrated in vacuo until precipitation was observed. The mixture was heated to reflux, and minimum *n*-hexane was added to redissolve all the precipitate. The concentrated clear solution stored at room temperature provided high-quality crystals of **7** (crude yield: 6.89g, 71.6%; Yield of the first batch of pure crystalline material: 1.06 g, 11%). ^{1}H NMR (400 Hz, $CDCl_3$, δ), 1.38 (s., ^{t}Bu). ^{31}P{^{1}H} NMR (162 Hz, $CDCl_3$, δ), 135.9 (s.). ^{13}C NMR (100 Hz, $CDCl_3$, δ), 32.3 (s.), 52.7 (s.). m.p. = 112-118 °C (decomposition). MS (EI) m/z: 1321.55 $[M+H]^{+}$.

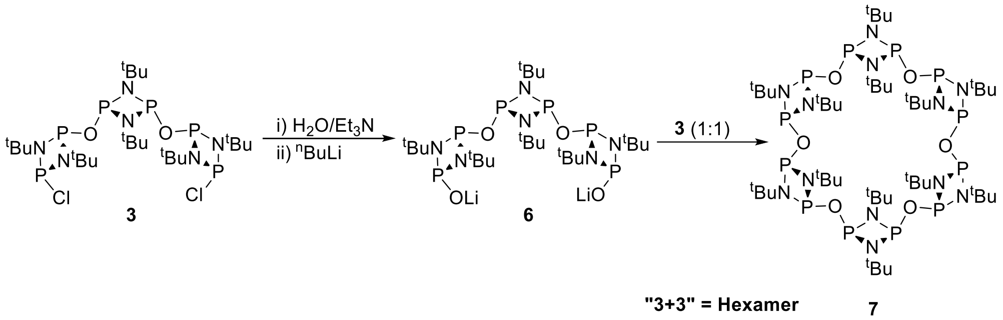

Supplementary Scheme S5

1.6 Synthesis of {(μ-O)[P(μ-N^{t}Bu)]$_2$}$_4$ (8) (Supplementary Scheme S6)

Compound **1** was dissolved in THF and slowly added to *in situ* generated **6** in THF at -78 °C dropwise in a 1:1 ratio. The reaction mixture was allowed to warm back to room temperature and stirred for overnight. The solvent of the solution was removed under vacuum and extracted with 30 mL *n*-hexane and filtered through Celite (P3). The reaction mixture was characterized by *in situ* ^{31}P NMR and showed a singlet at δ ~175.0 ppm. The solvent of the solution was further removed under vacuum and the solid was dissolved in distilled ACN, the HRMS spectrum revealed a peak at 881.37 $[M+H]^{+}$. The data obtained for **8** is consistent with the previously reported data for the {(μ-O)[P(μ-N^{t}Bu)]$_2$}$_4$ tetramer (*Dalton Trans.* 1293-1296 (2009). DOI: 10.1039/b900268).

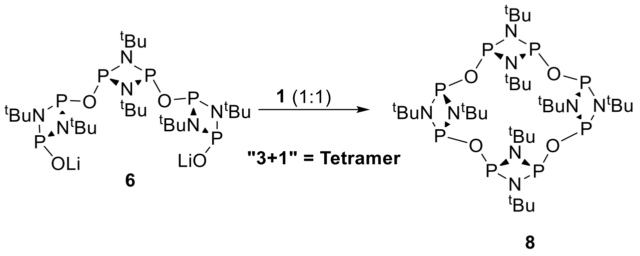

Supplementary Scheme S6

2 Characterisation of Compounds

2.1 NMR Spectra

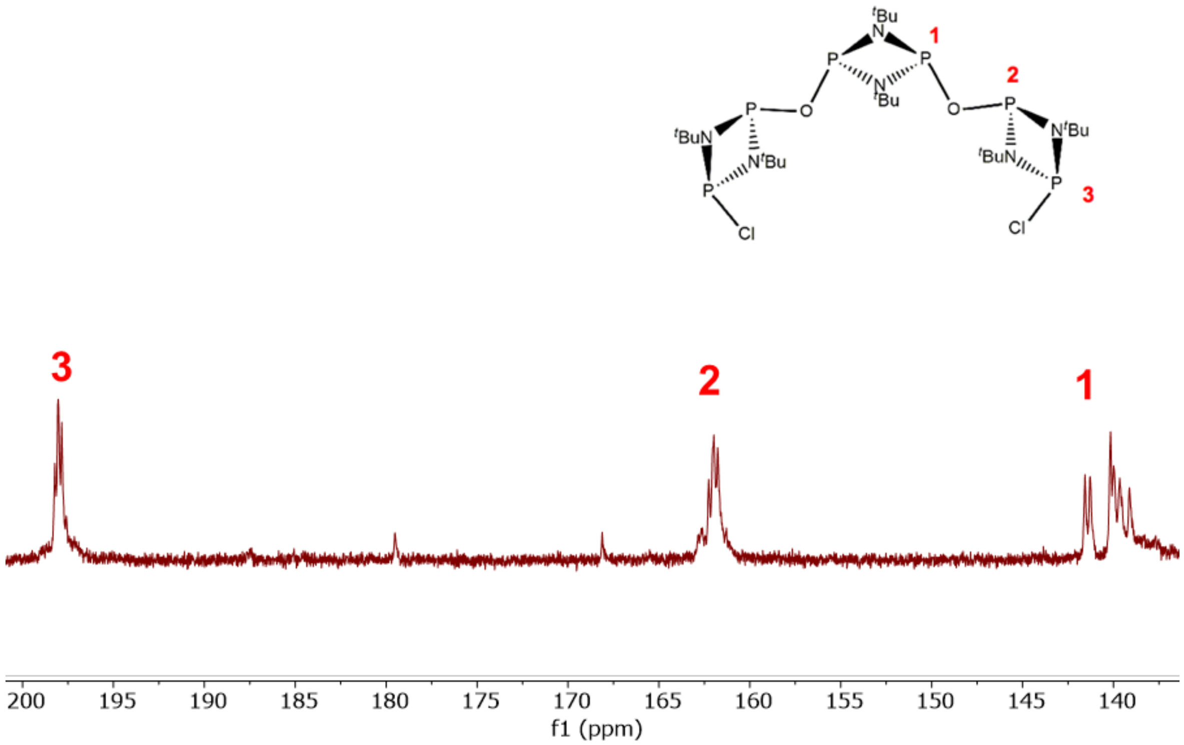

Supplementary Figure S1: *In situ* $^{31}P\{^{1}H\}$ NMR spectrum of $\{P(\mu\text{-}N^{t}Bu)\}_2(\mu\text{-}O)_2[\{P(\mu\text{-}N^{t}Bu)\}_2PCl]_2$ (**3**) in THF.

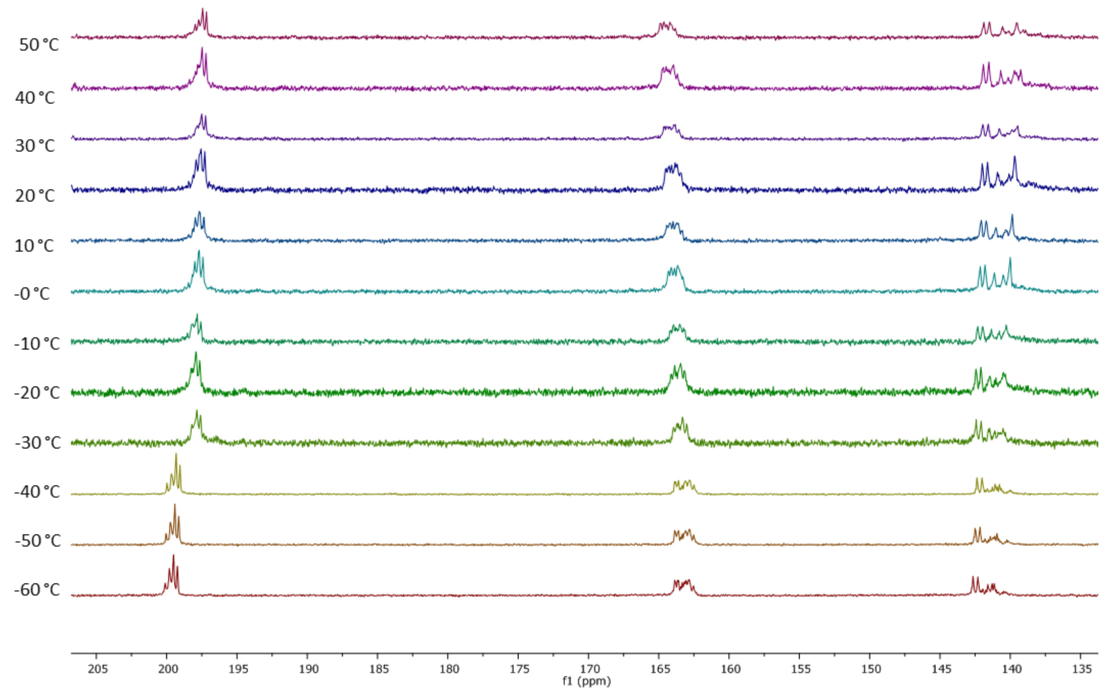

Supplementary Figure S2: Variable temperature $^{31}P\{^{1}H\}$ NMR spectrum of $\{P(\mu\text{-}N^{t}Bu)\}_2(\mu\text{-}O)_2[\{P(\mu\text{-}N^{t}Bu)\}_2PCl]_2$ (**3**) in THF.

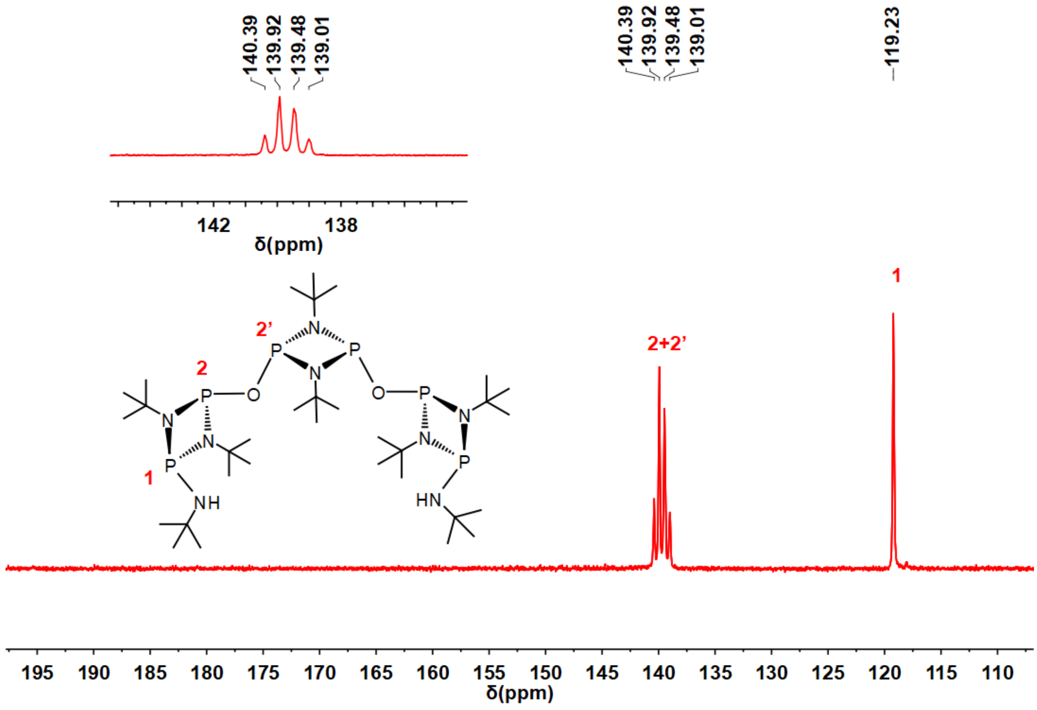

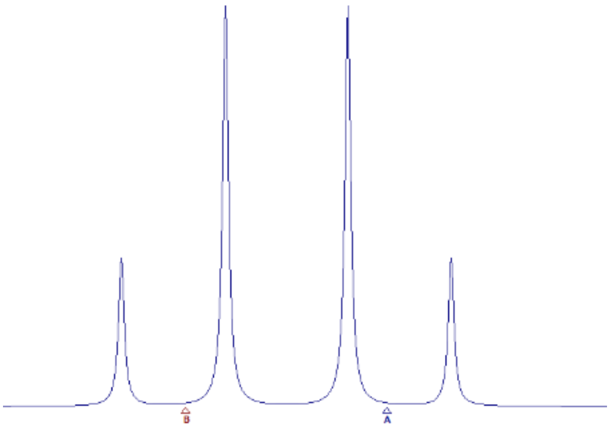

Supplementary Figure S3: $^{31}P\{^{1}H\}$ NMR spectrum of $\{P(\mu\text{-}N^{t}Bu)\}_2(\mu\text{-}O)_2[P(\mu\text{-}NtBu)2PN(H)^{t}Bu]_2$ (**4**) in THF (top) and simulated ^{31}P NMR spectra of the internal phosphorus atoms of **4** using WINDNMR (parameters = $\Delta\upsilon$ = 147 Hz, J = 76 Hz, FWHM = 0.5 Hz) (bottom).

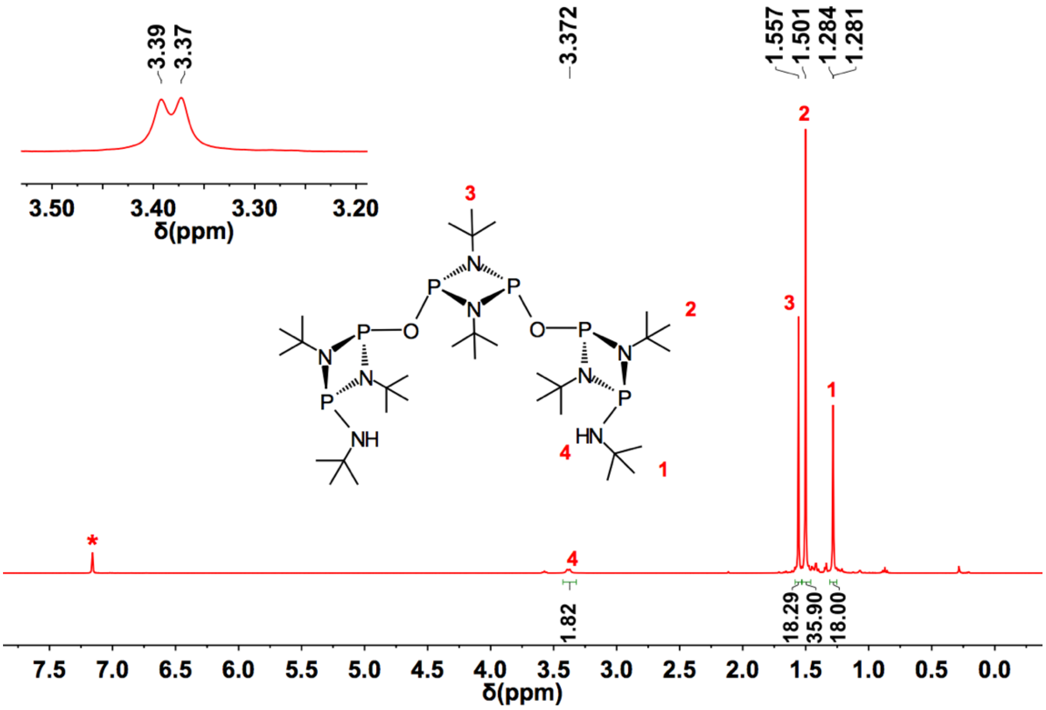

Supplementary Figure S4: ^{1}H NMR spectrum of $\{P(\mu\text{-}N^{t}Bu)\}_2(\mu\text{-}O)_2[\{P(\mu\text{-}N^{t}Bu)\}_2PNH^{t}Bu]_2$ (**4**) in C_6D_6.

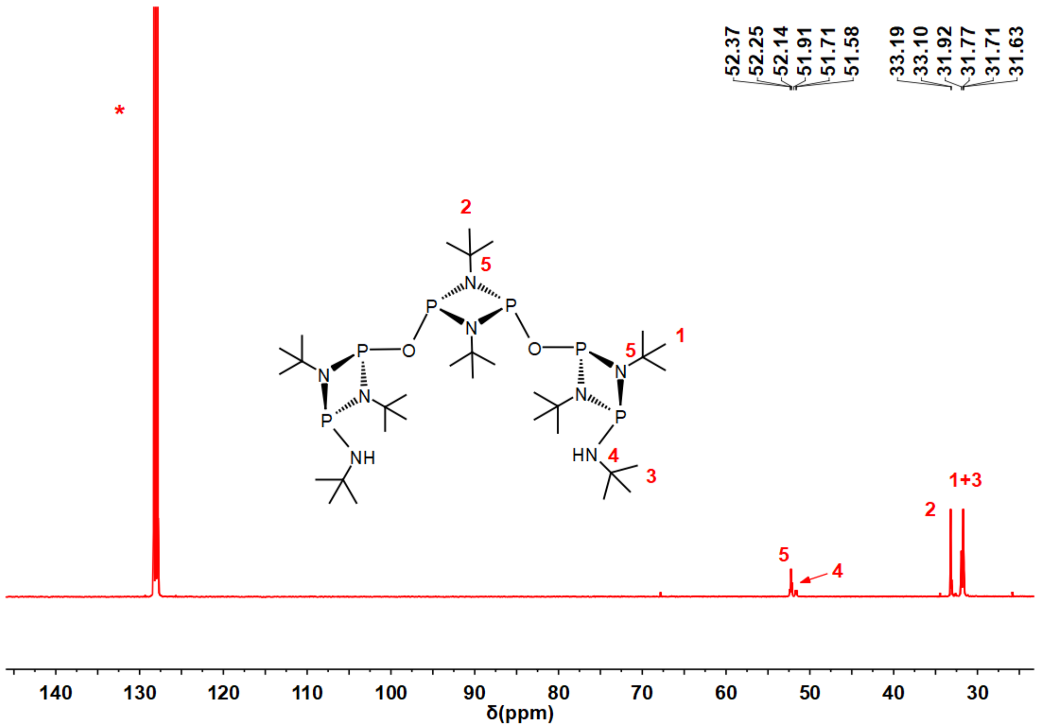

Supplementary Figure S5: ^{13}C NMR spectrum of {P(μ-N^{t}Bu)}$_2$(μ-O)$_2$[{P(μ-N^{t}Bu)}$_2$PNH^{t}Bu]$_2$ (**4**) in C_6D_6.

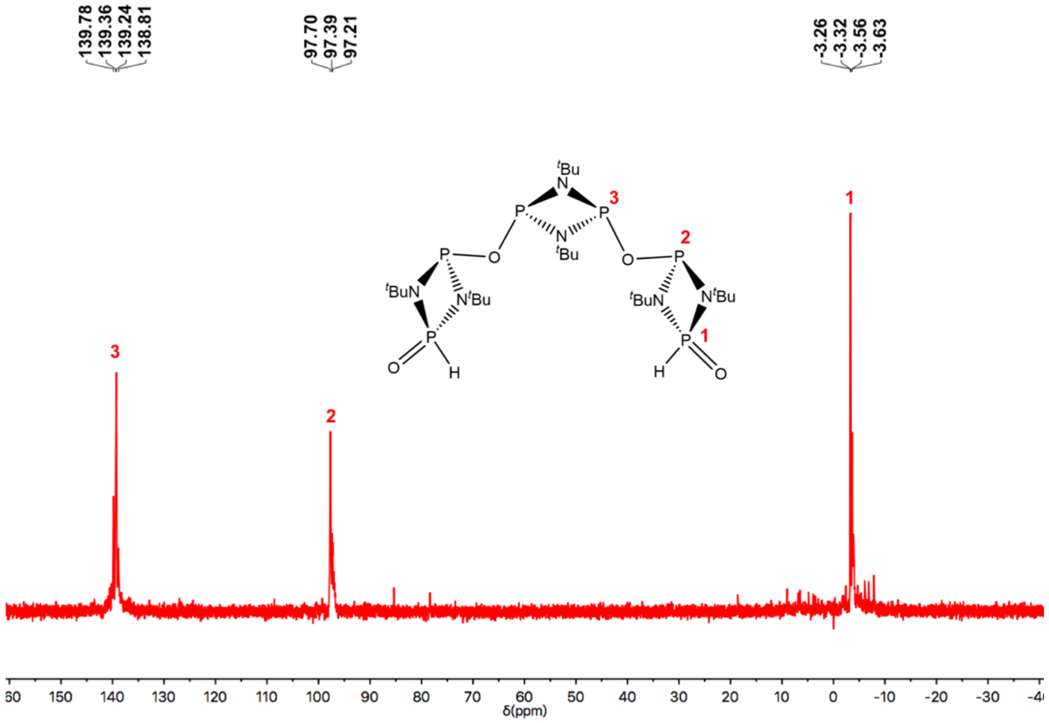

Supplementary Figure S6: *In situ* ^{31}P{^{1}H} spectrum of P(μ-N^{t}Bu)}$_2$(μ-O)$_2$[{P(μ-N^{t}Bu)}$_2$P(=O)H]$_2$ (**5**) in THF.

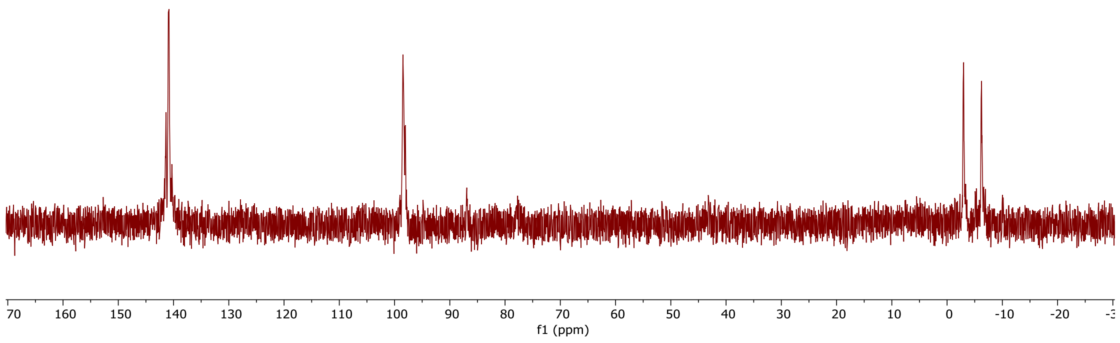

Supplementary Figure S7: *In situ* ^{31}P (spectrum of P(μ-N^{t}Bu)}$_2$(μ-O)$_2$[{P(μ-N^{t}Bu)}$_2$P(=O)H]$_2$ (**5**) in THF.

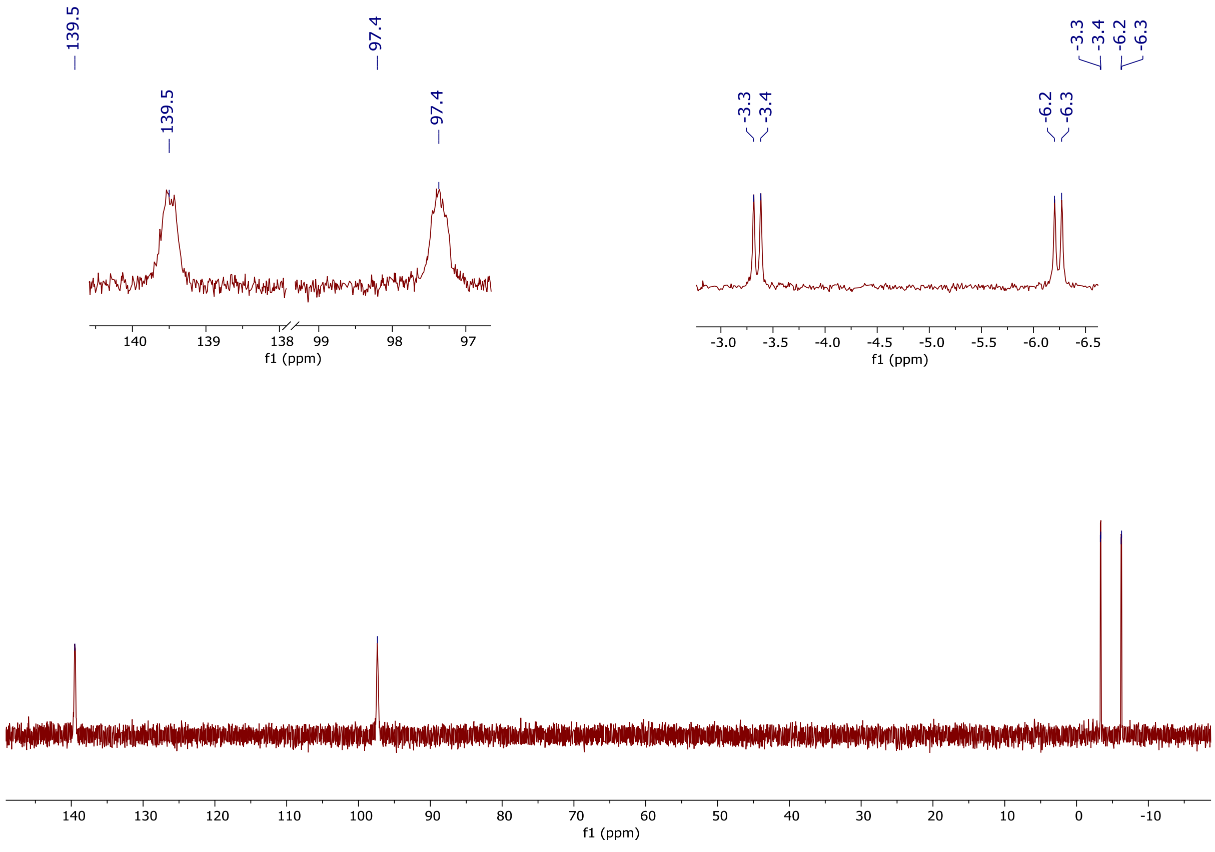

Supplementary Figure S8: H-coupled ^{31}P spectrum of P(μ-N^{t}Bu)}$_2$(μ-O)$_2$[{P(μ-N^{t}Bu)}$_2$P(=O)H]$_2$ (**5**) in C_6D_6.

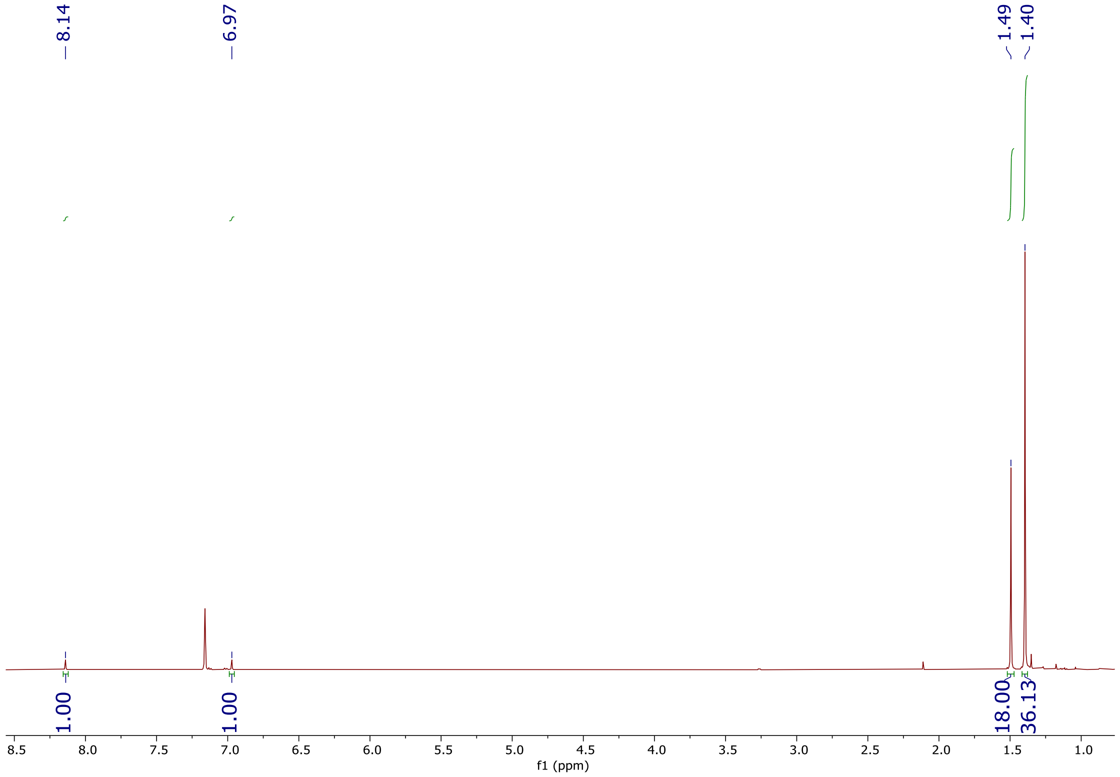

Supplementary Figure S9: ^{1}H NMR of P(μ-N^{t}Bu)}$_2$(μ-O)$_2$[{P(μ-N^{t}Bu)}$_2$P(=O)H]$_2$ (**5**) in C_6D_6.

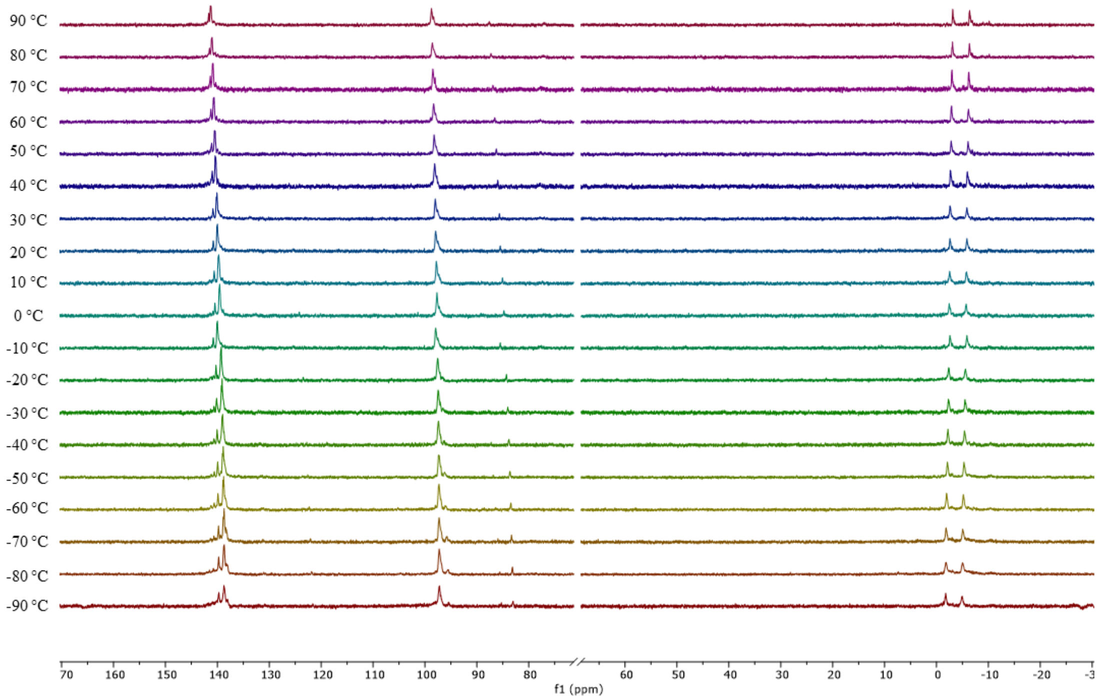

Supplementary Figure S10: Variable temperature ^{31}P NMR spectrum of P(μ-N^{t}Bu)}$_2$(μ-O)$_2$[{P(μ-N^{t}Bu)}$_2$P(=O)H]$_2$ (**5**) in toluene-d_8.

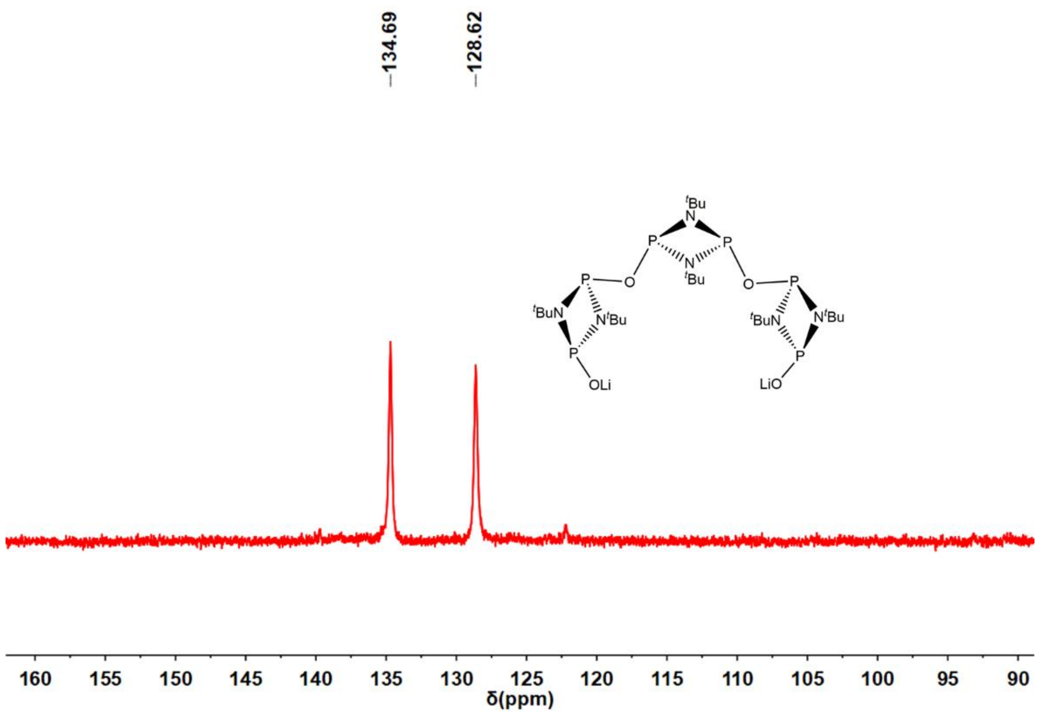

Supplementary Figure S11: *In situ* ^{31}P{^{1}H} NMR spectrum of {P(μ-N^{t}Bu)}$_2$(μ-O)$_2$[{P(μ-N^{t}Bu)}$_2$POLi]$_2$ (**6**) in THF.

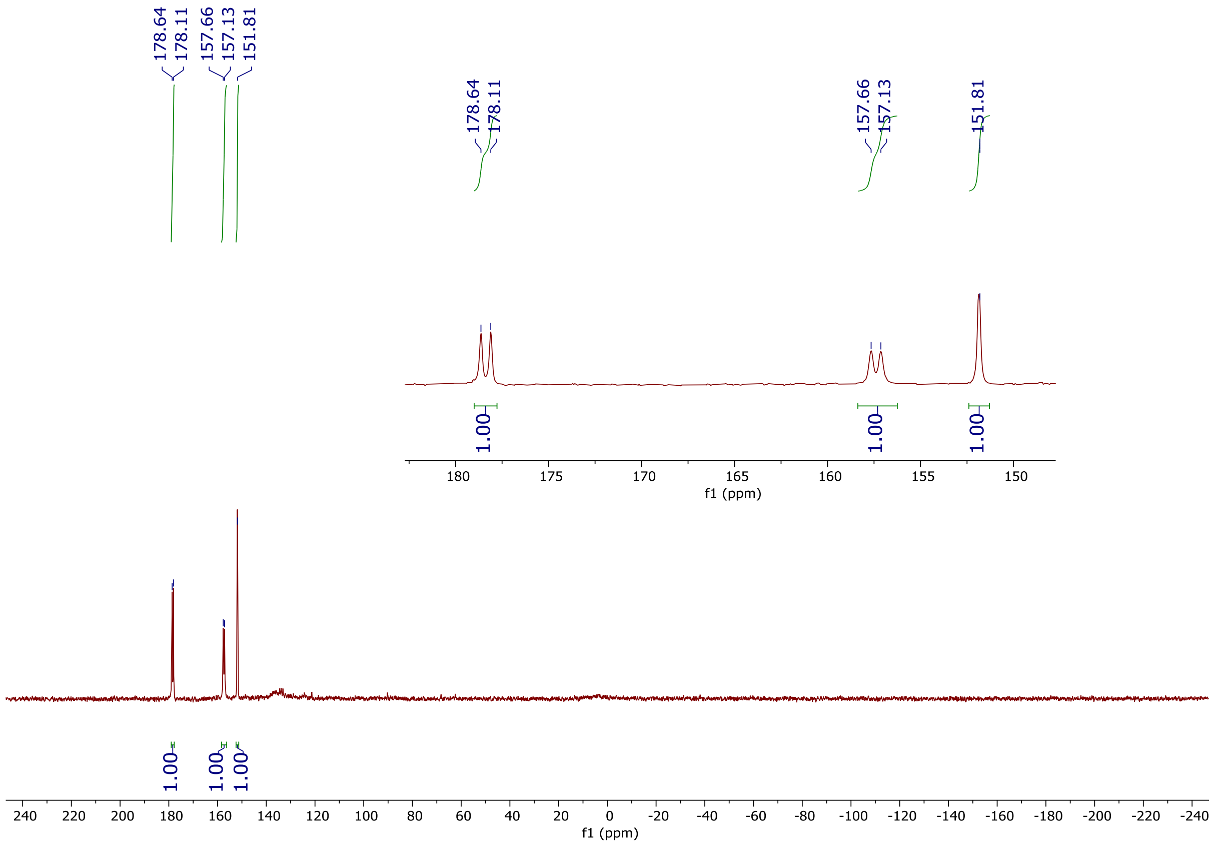

Supplementary Figure S12: *In situ* ^{31}P{^{1}H} NMR spectrum of {P(μ-N^{t}Bu)}$_2$(μ-O)$_2$[{P(μ-N^{t}Bu)}$_2$PONa]$_2$ (**6b**) in toluene.

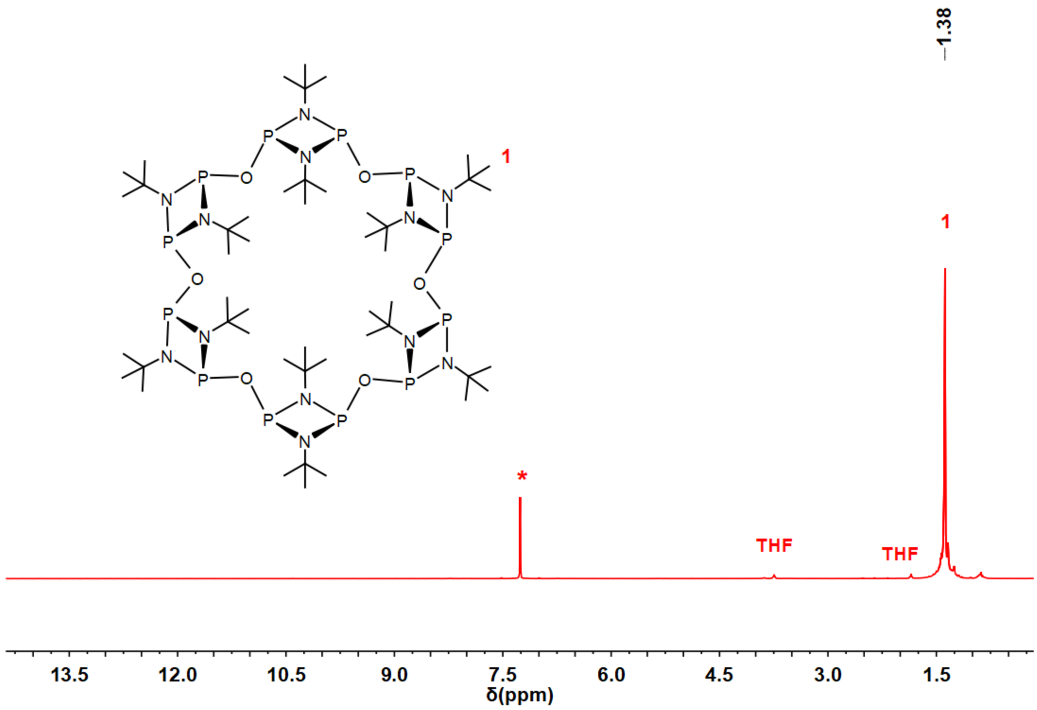

Supplementary Figure S13: ^{1}H NMR spectrum of $\{(\mu\text{-O})[P(\mu\text{-N}^t\text{Bu})]_2\}_6$ (**7**) in C_6D_6.

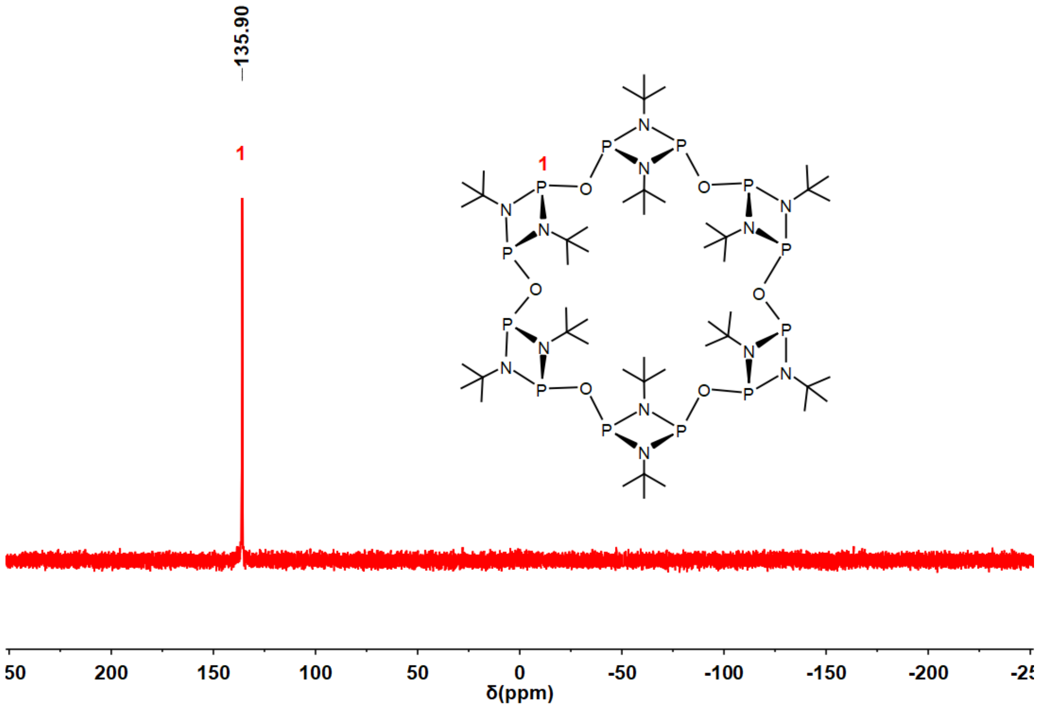

Supplementary Figure 14: $^{31}P\{^{1}H\}$ NMR spectrum of $\{(\mu\text{-O})[P(\mu\text{-N}^t\text{Bu})]_2\}_6$ (**7**) in C_6D_6.

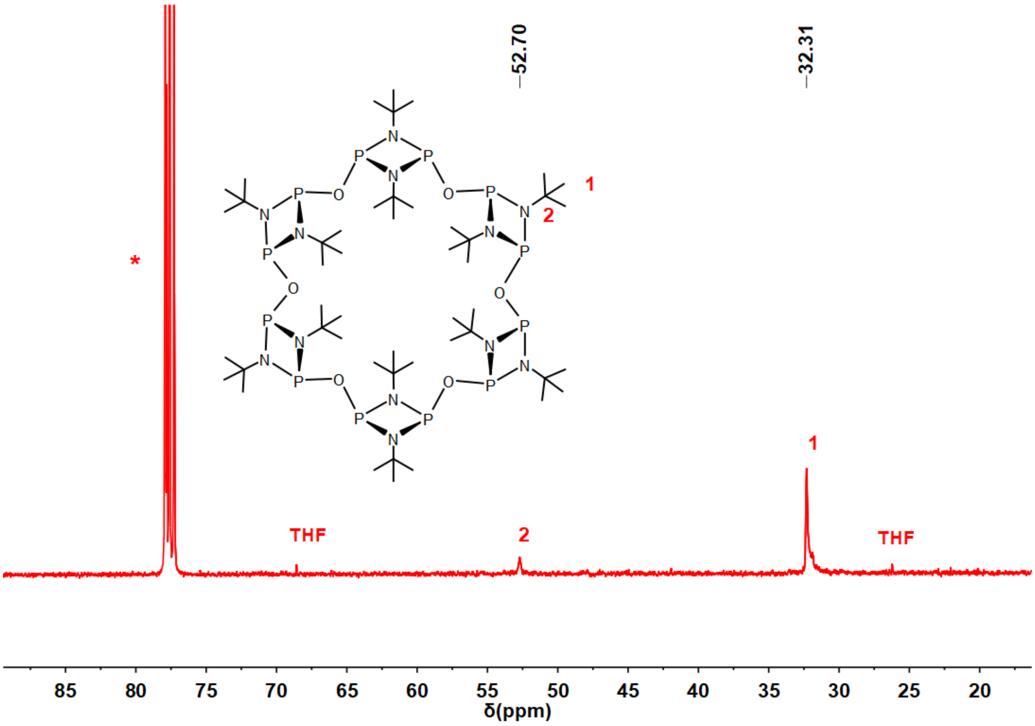

Supplementary Figure S15: ^{13}C NMR spectrum of {(μ-O)[P(μ-N^{t}Bu)]$_2$}$_6$ (**7**) in C_6D_6.

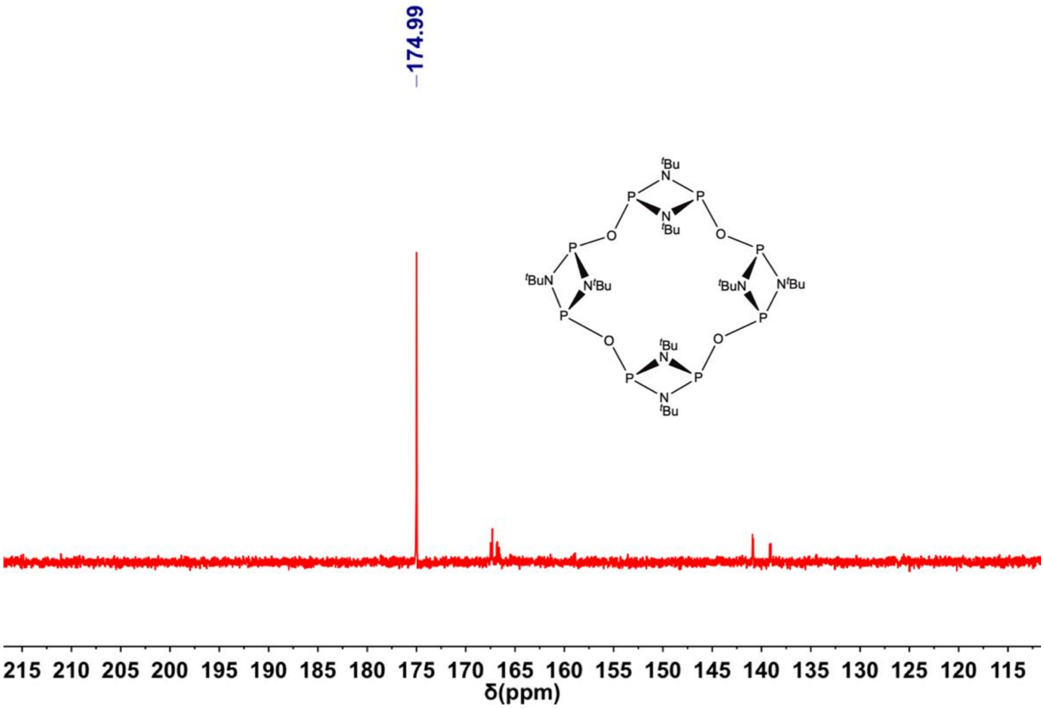

Supplementary Figure S16: *In situ* ^{31}P NMR spectrum of {(μ-O)[P(μ-N^{t}Bu)]$_2$}$_4$ (**8**).

2.2 FTIR and HRMS spectra

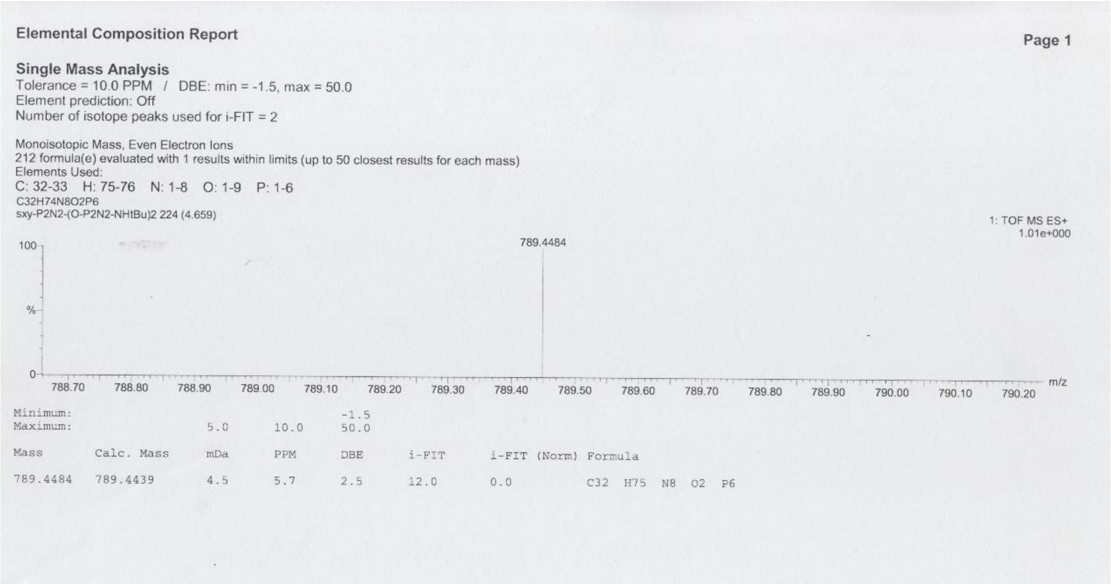

Supplementary Figure S17: HRMS spectrum of $\{P(\mu\text{-N}^t\text{Bu})\}_2(\mu\text{-O})_2[\{P(\mu\text{-N}^t\text{Bu})\}_2\text{PNH}^t\text{Bu}]_2$ (**4**).

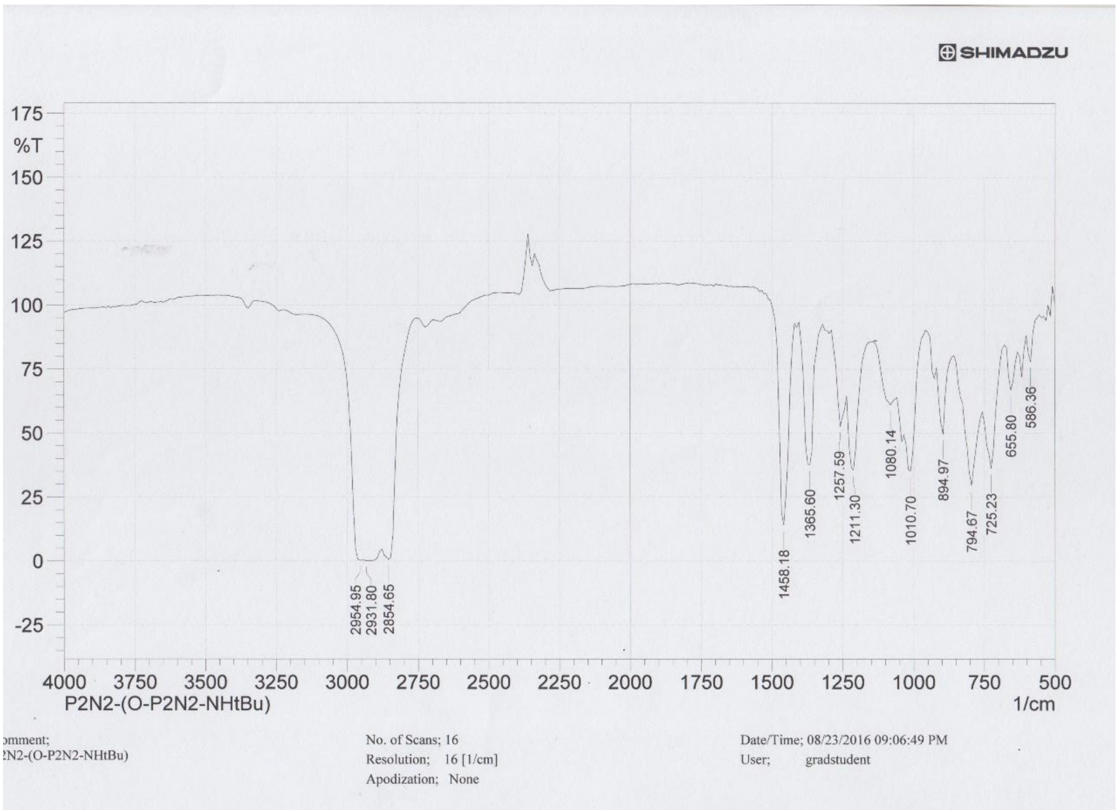

Supplementary Figure S18: IR spectrum of $\{P(\mu\text{-N}^t\text{Bu})\}_2(\mu\text{-O})_2[\{P(\mu\text{-N}^t\text{Bu})\}_2\text{PNH}^t\text{Bu}]_2$ (**4**).

Elemental Composition Report Page 1

Single Mass Analysis
Tolerance = 10.0 PPM / DBE: min = -1.5, max = 50.0
Element prediction: Off
Number of isotope peaks used for i-FIT = 3

Monoisotopic Mass, Even Electron Ions
3 formula(e) evaluated with 1 results within limits (up to 50 closest results for each mass)
Elements Used:
C: 24-25 H: 56-57 N: 5-6 O: 3-4 P: 5-6 S: 0-2

C42H67N4O4P4
TRIMEROH 82 (1.815) 1: TOF MS ES+ 1.01e+000

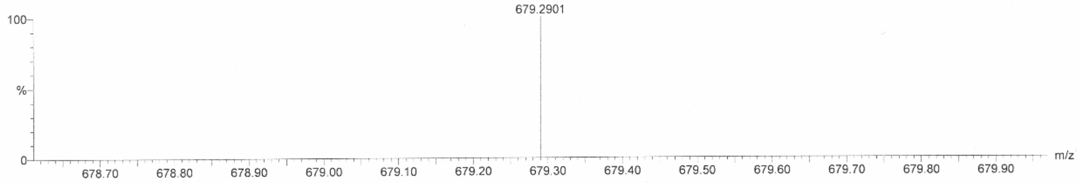

Minimum:				-1.5			
Maximum:		5.0	10.0	50.0			
Mass	Calc. Mass	mDa	PPM	DBE	i-FIT	i-FIT (Norm)	Formula
679.2901	679.2867	3.4	5.0	2.5	13.9	0.0	C24 H57 N6 O4 P6

Supplementary Figure S19: HRMS spectrum of {P(μ-N^{t}Bu)}$_2$(μ-O)$_2$[{P(μ-N^{t}Bu)}$_2$ P(=O)H]$_2$ (**5**).

Elemental Composition Report Page 1

Single Mass Analysis
Tolerance = 10.0 PPM / DBE: min = -1.5, max = 50.0
Element prediction: Off
Number of isotope peaks used for i-FIT = 3

Monoisotopic Mass, Even Electron Ions
3 formula(e) evaluated with 1 results within limits (up to 50 closest results for each mass)
Elements Used:
C: 24-25 H: 54-55 N: 5-6 O: 3-4 S: 0-2 P: 5-6 Li: 1-2

C42H67N4O4P4
TRIMEROLi 5 (0.120) 1: TOF MS ES+ 3.18e+000

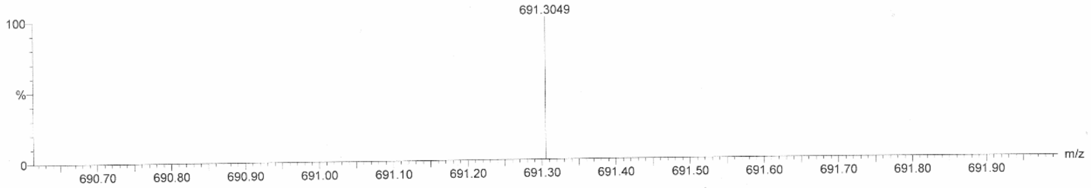

Minimum:				-1.5			
Maximum:		5.0	10.0	50.0			
Mass	Calc. Mass	mDa	PPM	DBE	i-FIT	i-FIT (Norm)	Formula
691.3049	691.3031	1.8	2.6	2.5	10.5	0.0	C24 H55 N6 O4 P6 Li2

Supplementary Figure S20: HRMS spectrum of {P(μ-N^{t}Bu)}$_2$(μ-O)$_2$[{P(μ-N^{t}Bu)}$_2$POLi]$_2$ (**6**).

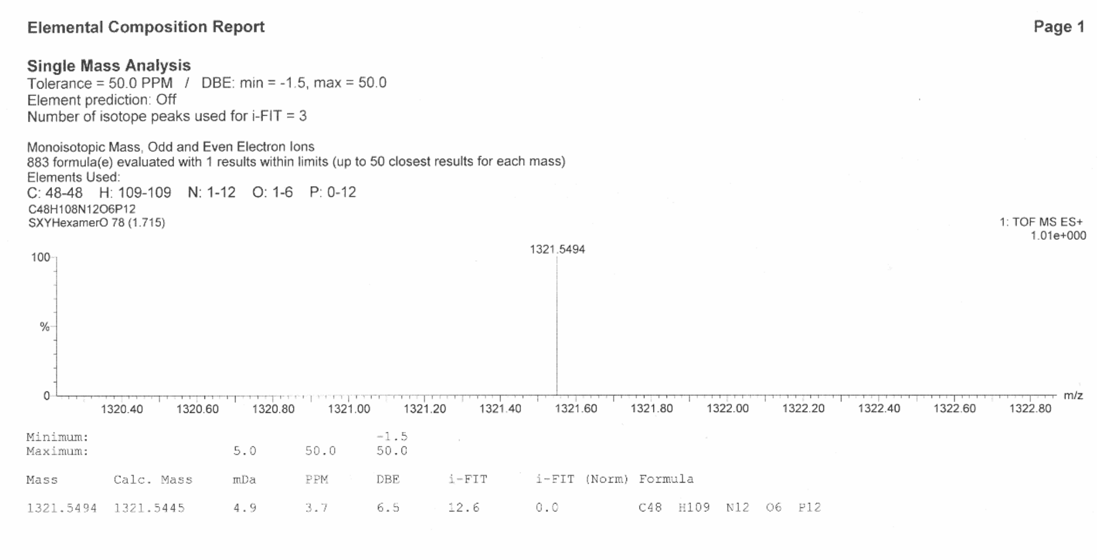

Supplementary Figure S21: HRMS spectrum of {(μ-O)[P(μ-N^{t}Bu)]$_2$}$_6$ (**7**)

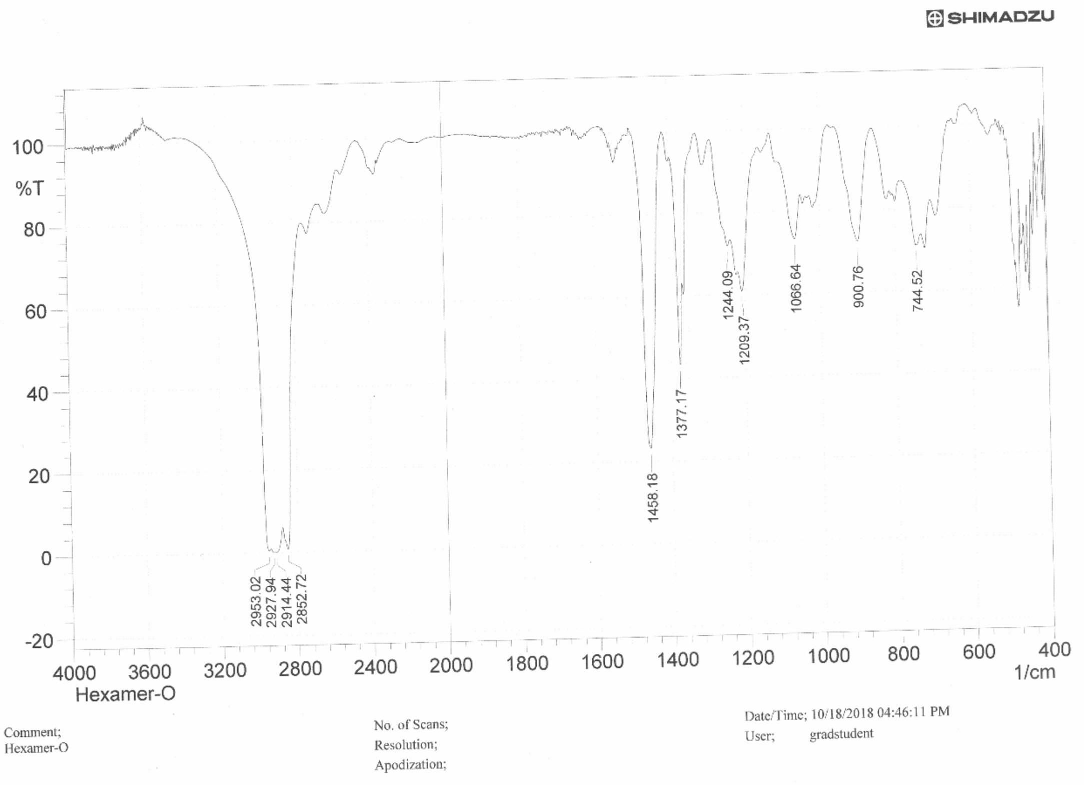

Supplementary Figure S22: IR spectrum of {(μ-O)[P(μ-N^{t}Bu)]$_2$}$_6$ (**7**).

Elemental Composition Report Page 1

Single Mass Analysis
Tolerance = 20.0 PPM / DBE: min = -1.5, max = 50.0
Element prediction: Off
Number of isotope peaks used for i-FIT = 3

Monoisotopic Mass, Even Electron Ions
1 formula(e) evaluated with 1 results within limits (up to 50 closest results for each mass)
Elements Used:
C: 32-33 H: 72-73 N: 7-8 O: 3-4 P: 7-8
C32H72N8O4P8
sxytetramer 89 (1.868) 1: TOF MS ES+ 1.01e+000

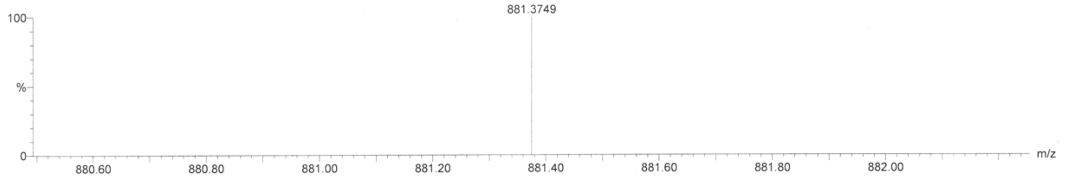

Minimum:				-1.5			
Maximum:		5.0	20.0	50.0			
Mass	Calc. Mass	mDa	PPM	DBE	i-FIT	i-FIT (Norm)	Formula
881.3749	881.3656	9.3	10.6	4.5	12.8	0.0	C32 H73 N8 O4 P8

Supplementary Figure S23: HRMS spectrum of {(μ-O)[P(μ-N^{t}Bu)]$_2$}$_4$ (**8**).

2.3 X-ray Analyses

Crystallographic Analyses. Diffraction-quality crystals were obtained by slow evaporation of solvent from solutions in *n*-hexane toluene solvent mixtures at room temperature. The crystals were mounted onto quartz fibers, and the X-ray diffraction intensity data were measured at 103 K with a Bruker Kappa diffractometer equipped with a CCD detector, employing Mo K α radiation (λ = 0.71073 Å), with the SMART suite of programs.[3] All data were processed and corrected for Lorentz and polarization effects with SAINT and for absorption effects with SADABS.[4] Structural solution and refinement were carried out with the SHELXTL suite of programs.[5] The structures were solved by direct methods or Patterson maps to locate the heavy atoms, followed by difference maps for the light, non-hydrogen atoms. All non-hydrogen atoms were refined with anisotropic thermal parameters.

Supplementary Table S1. X-Ray data of compounds **4**, **5** and **7.**

	**4**	**5**	**7**
Empirical formula	$C_{32}H_{74}N_8O_2P_6$	$C_{24}H_{56}N_6O_4P_6$	$C_{48}H_{108}N_{12}O_6P_{12}$
Formula weight	788.81	678.57	1321.10
Crystal system	Orthorhombic	Monoclinic	Monoclinic
Space group	P n m a	P 2 1/c	C 1 2/c 1
a/ Å	20.0290(5)	9.7183(19)	21.7911(18)
b /Å	23.3627(7)	41.855(5)	17.0239(18)
c/ Å	9.6838(3)	9.9893(12)	21.1950(19)
α/°	90	90	90
β/°	90	117.210(7)	106.148(6)
γ/°	90	90	90
Volume/ Å^3	4531.4(2)	3613.6(10)	7552.5(12)
Z	4	4	4
ρ (Calc)/Mg.m^{-3}	1.156	1.247	1.162
Absorp. Coeff./ mm^{-1}	0.273	0.334	0.316
F(000)	1712	1456	2832
Crystal Size/ mm^3	0.04 x 0.10 x 0.22	0.02 x 0.12 x 0.20	0.04 x 0.06 x 0.22
Θ range/ °	1.74 to 27.92	2.4 to 28.6	1.54 to 25.32
Index range	1.74 to 27.92<br>-30<=k<=30<br>-8<=l<=12	-12<=h<=13<br>-56<=k<=47<br>-10<=l<=12	-26<=h<=26<br>-20<=k<=20<br>-25<=l<=21
Refl. collected	80110	32131	34579
Indep. Refns. (R_{int})	5558 (0.0876)	9188 (0.099)	6846 (0.2015)
Completeness to Θ =	99.9 %	99.1%	99.0 %
Absorp. Corr.	multi-scan	multi-scan	multi-scan
Max., min., transmission	0.9890, 0.9420	0.94, 0.99	0.9870, 0.9340
Refinement Method	Full-matrix least-squares on F^2	Full-matrix least-squares on F^2	Full-matrix least-squares on F^2
Data/ restraint/parameters	5558 / 18 / 354	9188 / 247 / 468	6846 / 0 / 370
Goodness-of-fit on F^2	1.066	1.031	0.979
Final R indices [I>2σ(I)]	R1 = 0.0513, wR2 = 0.1127	R1 = 0.0586, wR2 = 0.1143	R1 = 0.0911, wR2 = 0.1949
R indices (all data)	R1 = 0.0790<br>wR2 = 0.1274	R1 = 0.1014<br>wR2 = 0.1352	R1 = 0.2541<br>wR2 = 0.2670
Largest diff. peak and hole/ e. Å^{-3}	0.408, -0.393	0.37, -0.40	0.502, -0.516

*Data in common: T = 103(2) K, λ = 0.71073 Å

In 4, one of the *tert*-butyl groups (C5, C6, C7, C8) was disordered over two positions with an occupancy of ~ 1:1. The atoms were refined without using any restraints (SIMU, ISOR) or constraints. In addition to it, one of the P-N group (P1, N5, C17, C18, C19, C20) was also disordered over two positions with about 50% occupancy in each site. The atoms were also refined without any constraints or restraints.

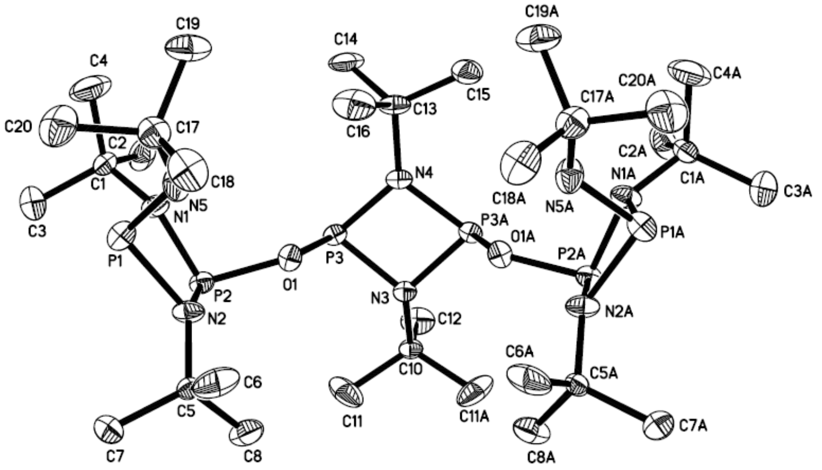

Donor--- ....Acceptor	D – H(Å)	H...A(Å)	D...A(Å)	D - H...A(°)	Symmetry Code
C2 -- H2C.. P2	0.980	2.780	3.150	103.0	Intra
C3 -- H3C.. P1	0.980	2.710	3.098	104.0	Intra
C4 -- H4A.. P1	0.980	2.790	3.177	104.0	Intra
C7A-- H7A2.. O1	0.980	2.580	3.193	120.0	Intra
C11-- H11C.. P3	0.980	2.820	3.197	104.0	Intra
C19A-- H19D.. P1A	0.980	2.860	3.185	100.0	Intra

Supplementary Figure S24. ORTEP structure of **4** (drawn with 50% probability, top). H-atoms and the disordered atoms are removed for clarity, selected Bond Lengths [Å] and Angles [deg]: N(1)–P(1) 1.676(4), N(2)–P(1) 1.808(4), N(2)–P(1) 1.681(6), N(2)–P(2) 1.692(2), N(1)–P(2) 1.705(2), O(1)–P(2) 1.6771(18), O(1)–P(3) 1.6447(17), N(3)–P(3) 1.7158(19), N(4)–P(3) 1.7018(19); N(1)-P(1)-N(2) 80.33(17), N(1)-P(2)-N(2) 82.94(10), P(2)-N(1)-P(1) 99.58(16), P(2)-N(2)-P(1) 95.03(15), P(2)-O(1)-P(3) 124.74(10). H atoms have been omitted for clarity. Table with selected hydrogen bonds interactions (bottom).

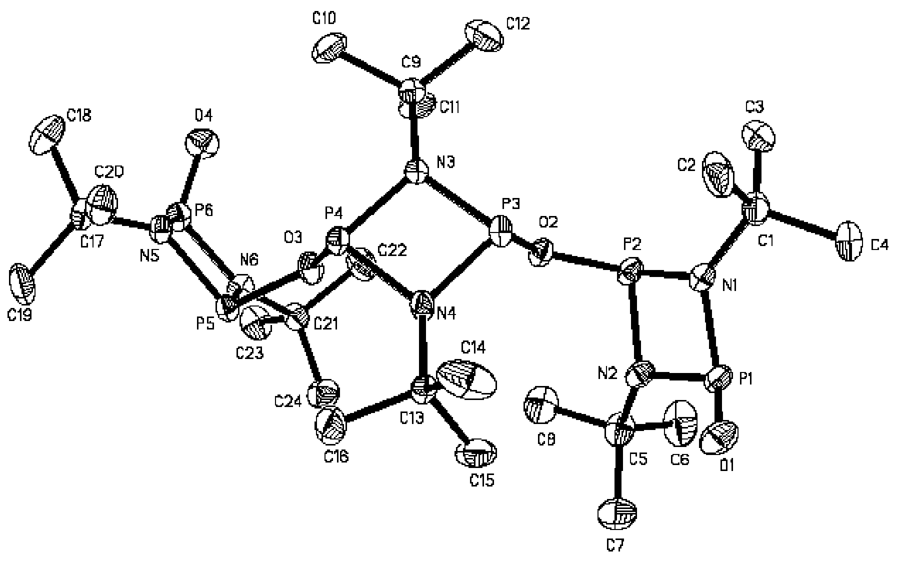

Donor--..Acceptor	D – H(Å)	H...A(Å)	D...A(Å)	D - H...A(°)	Symmetry Code
C8 – H8C.. P2	0.980	2.850	3.192	101.0	Intra
C11 – H11A.. O4	0.980	2.600	3.503	154.0	x,3/2-y,-1/2+z
C12 – H12B.. P3	0.980	2.860	3.196	101.0	Intra
C16—H16B.. O1	0.980	2.490	3.449	165.0	1-x,1-y,1-z
C24—H24C.. P5	0.980	2.830	3.152	100.0	Intra

Supplementary Figure S25. ORTEP structure of **5** (drawn with 50% probability, top). H-atoms and the disordered atoms are removed for clarity, selected Bond Lengths [Å] and Angles [deg]: N(1)–P(1) 1.710(6), N(2)–P(1) 1.712(6), N(2)–P(2) 1.718(6), N(1)–P(2) 1.688(6), O(1)–P(1) 1.655(5); N(1)-P(1)-N(2) 81.4(3), N(1)-P(2)-N(2) 81.8(3), P(2)-N(1)-P(1) 98.1(3), P(2)-N(2)-P(1) 96.9(3), P(1)-O(1)-P(6) 121.7(3). Table with selected hydrogen bonds interactions (bottom)

In **7**, the structure was solved and refined using the Bruker SHELXTL Software Package, using the space group C 1 2/c 1, with Z = 4 for the formula unit, C48H108N12O6P12. The final anisotropic full-matrix least-squares refinement on F2 with 370 variables converged at R1 = 9.11%, for the observed data and wR2 = 26.70% for all data. The goodness-of-fit was 0.979. The largest peak in the final difference electron density synthesis was 0.502 e-/Å3, and the largest hole was -0.516 e-/Å3 with an RMS deviation of 0.137 e-/Å3. 7 exhibits weak intra-molecular H-bonding (C3 -- H3A.. P2; C8 -- H8B.. P1; C11-- H11C.. P4; C18 -- H18C.. P5; C22-- H22C.. P6 and C23-- H23A.. O1)

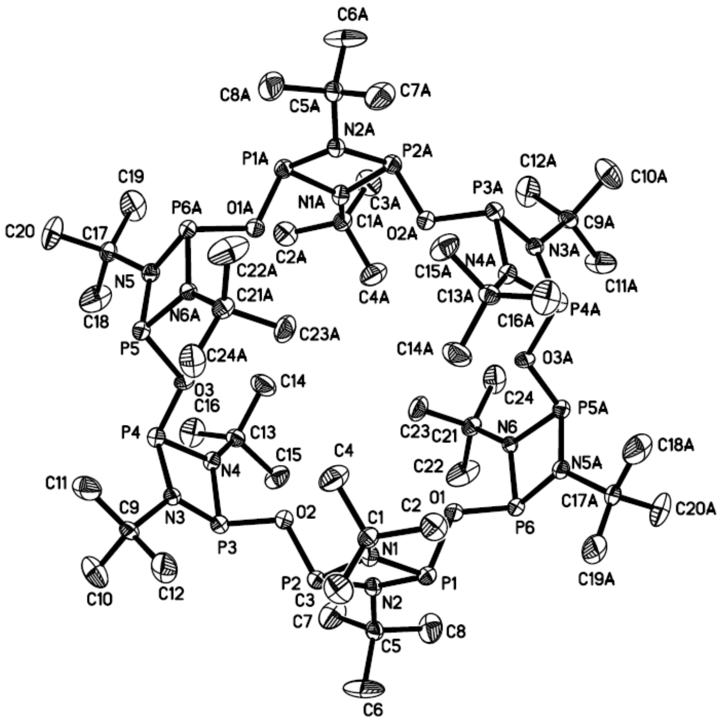

Donor--..Acceptor	D – H(Å)	H...A(Å)	D...A(Å)	D - H...A(°)	Symmetry Code
C3 -- H3A.. P2	0.970	2.870	3.273	106.0	Intra
C8 -- H8B.. P1	0.970	2.710	3.1472	108.0	Intra
C11-- H11C.. P4	0.970	2.800	3.129	101.0	Intra
C18 -- H18C.. P5	0.970	2.850	3.253	106.0	Intra
C19-- H19A.. P6	0.970	2.830	3.191	103.0	1-x,1-y,1-z
C22-- H22C.. P6	0.970	2.740	3.138	105.0	Intra
C23-- H23A.. O1	0.970	2.570	3.276	129.0	Intra
C24-- H24A.. P5	0.970	2.850	3.157	100.0	1-x,1-y,1-z

Supplementary Figure S26. ORTEP structure of **7** (drawn with 50% probability, top). H-atoms and the disordered atoms are removed for clarity, selected Bond Lengths [Å] and Angles [deg]: N(1)–P(1) 1.710(6), N(2)–P(1) 1.712(6), N(2)–P(2) 1.718(6), N(1)–P(2) 1.688(6), O(1)–P(1) 1.655(5); N(1)-P(1)-N(2) 81.4(3), N(1)-P(2)-N(2) 81.8(3), P(2)-N(1)-P(1) 98.1(3), P(2)-N(2)-P(1) 96.9(3), P(1)-O(1)-P(6) 121.7(3). Table with selected hydrogen bonds interactions (bottom)

3 Theoretical Studies – Supplementary Discussion.

Density functional theory (DFT) (ω-B91xD/6-31G(d,p))[6,7] calculation were performed on Gaussian 16.[8] The geometry and optimization energies on compound **7** and selected crown-ethers (*i.e.*, **18C6** and **21C7**) were computed to assess and compare the volume of their internal cavities.

The geometry optimization of **7**, shown in **Supplementary Figure S27**, display a highly symmetrical framework where the P_2N_2 rings adopt a puckered zig-zag arrangement – with respect of the main the main macrocycle plane (defined by the oxygen bridging atoms, see main text, **Figure 3**) – with the *tert*-butyl groups displaying an eclipsed conformation – presumably to reduce the steric strain imposed by the presence of twelve bulky tert butyl groups within the macrocycle backbone.

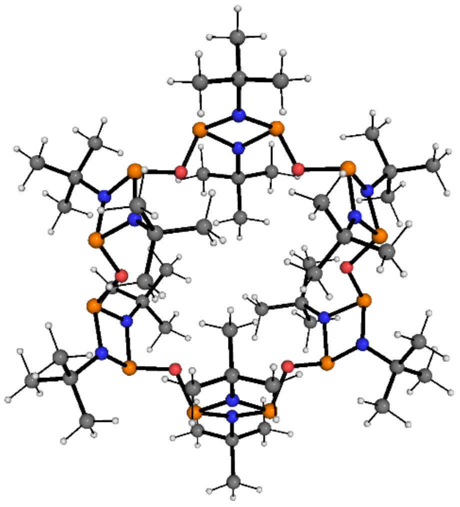

Supplementary Figure S27. Geometry optimization of **7** at (SMD, THF)-ω-B97xD/6-31G(d,p) level of theory.

The six P_2N_2 rings presents in **7** are bridged by six oxygen atoms determining a hexagonal polar cavity highly resembling that of crown-ethers. Therefore, further calculations were performed on **7**, **18C6** and **21C7** to assess their cavity sizes (see **Supplementary Figure S28**), which were in agreement the value obtained from the X-ray data obtained experimentally.

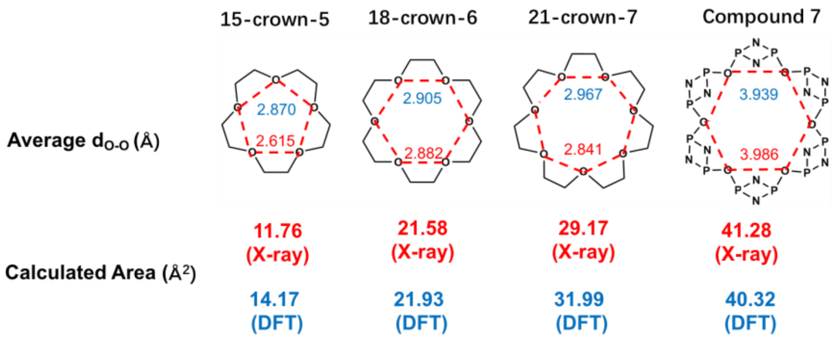

Supplementary Figure S28: Calculated inside area for different compounds

Moreover, due to the puckered zig-zag arrangement of the P_2N_2 rings, the six ^{t}butyl groups above and below the central hexagonal the plane, delimited by the bridging oxygen atoms, define a hypothetical irregular icosahedron with a calculated internal volume of 120 Å^3

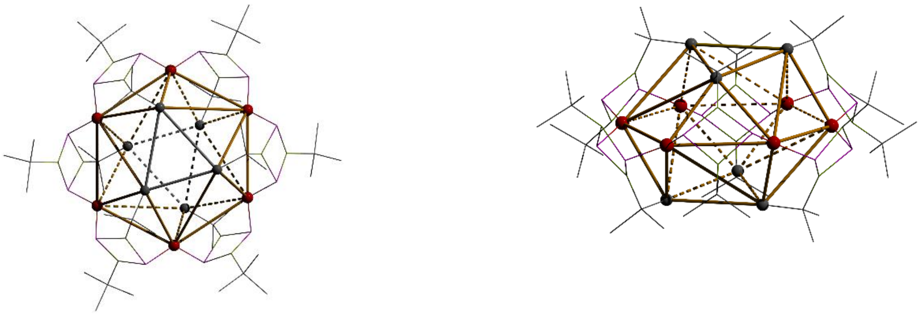

Top view **Side view**

Supplementary Figure S29. Top and side views of the of the internal volume defined an icosahedron determined by the oxygen atoms and the *tert*-butyl groups in **7.**

To illustrate the large size of the cavity, comparison with the volume of a Bukyball C60 are shown in figure S33. The volume of C60 slightly higher than the calculated cavity presents in **7** (~ 180 Å^3 and 120 Å^3, respectively). However, considering C60's diameter (~7 Å^2), hypothetically C60 could fit within the cyclophosphazane cavity as the calculated area delimited by the oxygen atom present in **7** is slightly larger (~ 38.48 Å^2 and ~ 40.32 Å^3 for C60 and **7**, respectively).

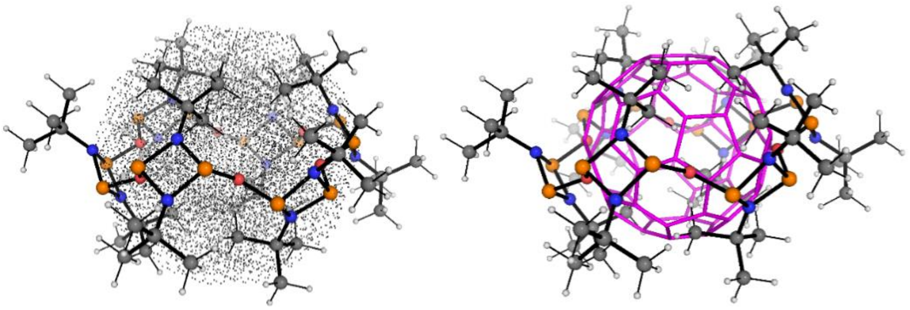

Supplementary Figure S30: Geometry optimization of **7** and buckyball C60 at ω-B91xD/6-31G(d,p) level of theory, C60 is represented as dots (left) and wires (right).

Regarding host-guest ability. it has been demonstrated that density functional theory (DFT) cyclophosphazane species result in good agreement between theoretical and experimental values,[26] we performed DFT calculations to assess the host capacity of **7** relative to the classic crown ethers **18C6** and **21C7** using K^+, $[NH_4]^+$, [1,1'-biphenyl]-2,2'-diamine, and [1,1'-biphenyl]-2,2'-diammonium as guest molecules.

The different interaction with K^+ cation was modelled for compounds **7**, **18C6** and **21C7**. Our calculations show that the interaction between the metal centre and the donor oxygen atoms in the studied crown ethers is almost constant (2.78-2.81Å, and 2.90-2.99 Å in **18C6** and **21C7**, respectively) – see **Supplementary Figures S31** and **S32**. In contrast, in **7** the interactions displayed are highly asymmetric (2.75-5.32 Å) due to the larger cavity present - see **Supplementary Figure S33**).

In the case of K^+ guest, both crown ethers display higher binding energies than **7** (*i.e.***,** -27.4 and -27.1 *vs* -13.7 kcal·mol^{-1}, with **18C6**, **21C7**, and **7**, respectively, **Supplementary Table S1**). Moreover, DFT data revealed that the [K^+·crown ether] adducts display comparably short (< 3 Å), relatively strong, $K^+\cdots O$ interactions across each structure (*i.e.* ranging from 2.78-2.81Å, and 2.90-2.99 Å in **18C6** and **21C7**, respectively) – see **Supplementary Figures S31** and **S32.** In contrast, **7** displays a broader range of $K^+\cdots O$ distances (2.75-5.32 Å, **Supplementary Figure S33**). Notably, the calculated binding energy for **7** is relatively high

considering the asymmetric nature of the $K^{+}\cdots O$ interactions and the presence of only two strong interactions below 3 Å (*cf.* six and seven short contacts in **18C6** and **21C7**, respectively).

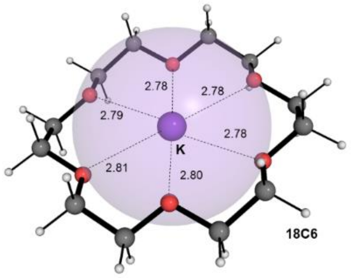

Supplementary Figure S31. Geometry optimization of **18C6** encapsulating a Potassium ion at ω-B91xD/6-31G(d,p) level of theory, selected distances in Angstrom.

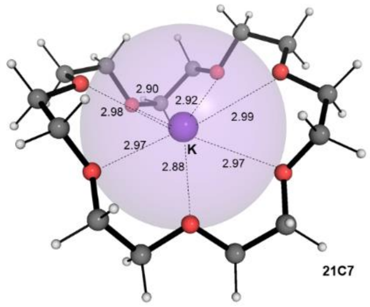

Supplementary Figure S32. Geometry optimization of **21C7** encapsulating a Potassium ion at ω-B91xD/6-31G(d,p) level of theory, selected distances in Angstrom.

a **b**

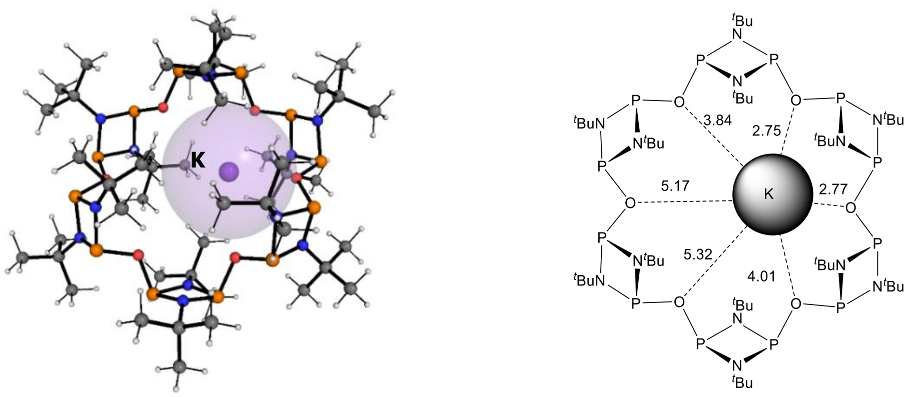

Compound 7

Supplementary Figure S33. Geometry optimization for compound **7** encapsulating a Potassium ion at ω-B91xD/6-31G(d,p) level of theory, selected distances in Angstrom.

Supplementary Table S1. Binding energy at ω-B91xD/6-31G(d,p) theory level for the **[X·K]**$^{+}$ host-guest adduct (**X= 18C6**, **21C7**, and **7**; and **K**$^{+}$**)**

	**18C6**	**21C7**	**7**
**Binding energy (G, kcal/mol)**	-27,4	-27,1	-13,7
**Average bond distance**	2.79 A	2.94 A	3.97

Following these studies, we assessed the host-guest properties of compound **7** compared with **18C6** and **21C7**, using cationic ammonium, neutral [1,1'-biphenyl]-2,2'-diamine and dicationic [1,1'-biphenyl]-2,2'-diaminium. The optimization was performed at ω-B91xD/6-31G(d,p) level of theory and single point calculations taking into account the solvent effects were performed on the optimized structure. The Gibbs energy of these interaction are shown in **Supplementary Tables S2** and **S3**.

When $[NH_4]^+$ is used a guest molecule, its adducts with **18C6** and **21C7** display three non-bonding hydrogen bonds (HB) (**Supplementary Figures S34a** and **S35a**), whereas only two HB interactions are present in **7** (Figures **S36a**). To compare different host-guest adducts, binding energy expressed relative to HB interaction number was calculated (**Supplementary Table S2**). Our assessment shows that the relative binding energy of **7** (*ca.* -8.6 kcal·mol^{-1} per interaction) is comparable to those displayed by both **18C6** and **21C7** crown ethers (*ca.* -9.7 kcal·mol^{-1}). Furthermore, non-covalent interaction (NCI) analyses clearly display two attractive host-guest interactions between the two of the NH present in $[NH_4]^+$ and two adjacent oxygen bridging atom in **7** (*vide infra*, **Supplementary Figure S37**).

Supplementary Table S2. Binding energy, and relative binding energy (*i.e.*, binding energy/number of hydrogen bonds) (in kcal·mol^{-1})**,** and average N-H···O distance in the [**X**·NH$_4$]$^+$ host-guest adduct (**X**= **18C6, 21C7**, and **7**)**. Calculated at** ω-B91xD/6-31G(d,p) level of theory

	18C6	21C7	7
**Bind. E (kcal/mol)**	-29.1	-29.2	-17.2
**Rel. Binding E) (kcal/mol)**	-9.7	-9.7	-8.6
**Average N-H···O distance (Å)**	1.8	1.9	1.7

a **b**

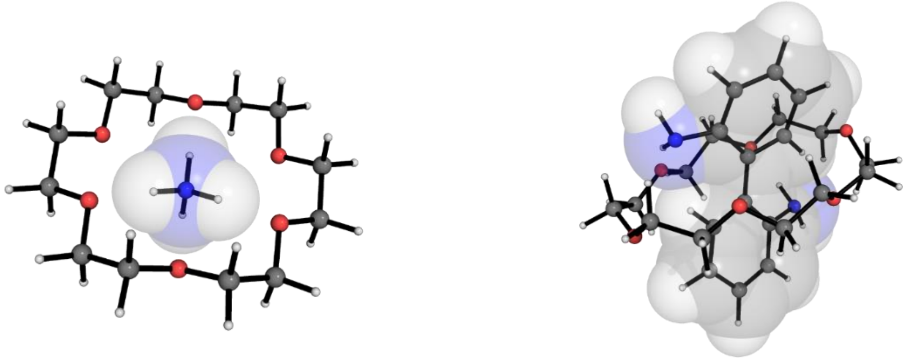

Supplementary Figure S34. Geometry optimization of **18C6** host-guest adduct with ammonium cation (a) and 1,1'-biphenyl]-2,2'-diamine (b) at (SMD)-ω-B97xD/6-31G(d,p) level of theory.

a **b**

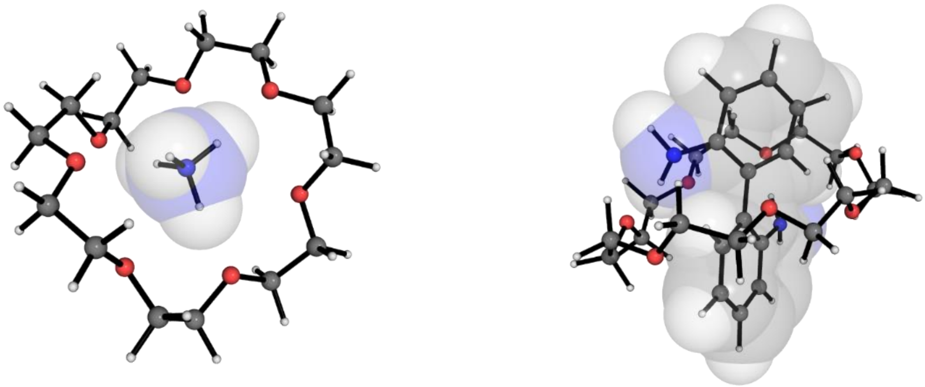

Supplementary Figure S35. Geometry optimization of **21C7** host-guest adduct with ammonium cation (a) and1,1'-biphenyl]-2,2'-diamine (b) at (SMD)-ω-B97xD/6-31G(d,p) level of theory.

a **b**

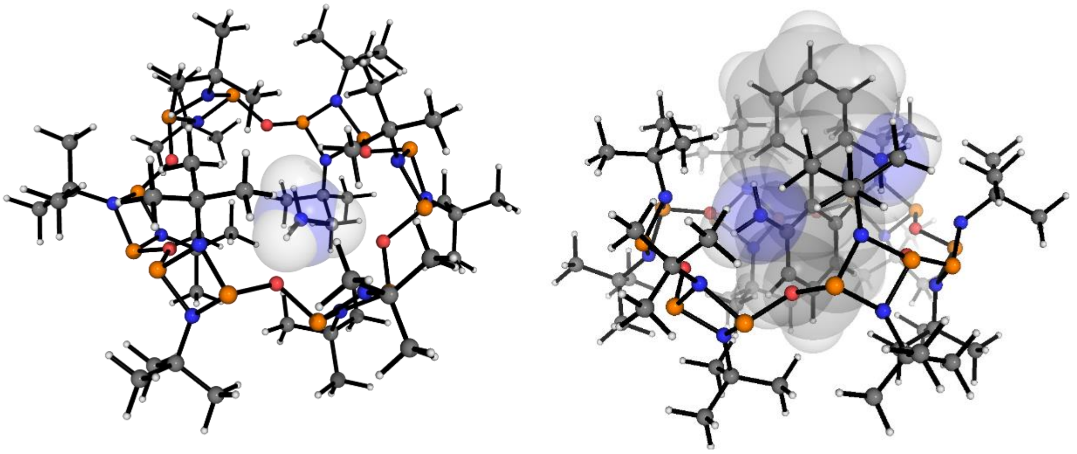

Supplementary Figure S36. Geometry optimization of **7** host-guest adduct with ammonium cation (a) and 1,1'-biphenyl]-2,2'-diamine (b) at (SMD)-ω-B97xD/6-31G(d,p) level of theory.

In the case of larger neutral and charged organic guests, we computed theoretical host-guest interactions for [1,1'-biphenyl]-2,2'-diamine and its di-protonated derivative (*i.e.*, [1,1'-biphenyl]-2,2'-diaminium cation) with **18C6**, **21C7** and **7**. Both crown ethers display unfavourable interaction with the organic guest as shown by the overall large increase in the calculated binding energy (+112.5, and +30.6 kcal·mol^{-1} for **18C6**, and **21C7**, respectively). In contrast, compound **7** displays a less unfavourable host-guest interaction with a calculated energy of 16.5 kcal·mol^{-1} with the diamine guest (see **Supplementary Table S3**).

Supplementary Table S3. Binding energy (in kcal·mol^{-1}) **for the** host-guest adducts [**X**·**L**]$^{+}$ host-guest adduct (**X= 18C6, 21C7, a**nd **7;** and **L=** 1,1'-biphenyl]-2,2'-diamine and 1,1'-biphenyl]-2,2'-diammonium). Calculated at ω-B91xD/6-31G(d,p) level of theory

	**18C6**	**21C7**	**7**
**1,1'-biphenyl]-2,2'-diamine**	112.5	30.6	16.5
**1,1'-biphenyl]-2,2'-diammonium**	94.1	-0.4	-26.4

When [1,1'-biphenyl]-2,2'-diamine is replaced by its protonated version (*i.e.*, [1,1'-biphenyl]-2,2'-diaminium cation) the differential host ability between **7** and the crown

ethers was more pronounced (**Supplementary Table S3** and **Supplementary Figures S34b-S36b**). Compound **7** shows a strong favourable interaction (-26.44 kcal·mol^{-1}), whereas for **18C6** the interaction is highly unfavourable (+94.07 kcal·mol^{-1}) and only slightly favourable for **21C7** (-0.38 kcal·mol^{-1}).

The differences displayed between the hosts is attributed to both the smaller cavity size present in **18C6**, and **21C7** crown ethers, and the presence of an amphiphilic cavity in **7**. Compound **7** comprises two well-defined zones, two non-polar regions comprising three tert-butyl groups each, sandwiching a central polar region defined by the oxygen atoms along the cavity equator. The amphiphilic nature of the macrocyclic cavity enables hosting large molecules comprising both polar and non-polar regions (such as [1,1'-biphenyl]-2,2'-diaminium). The presence of both polar and non-polar interactions is supported by NCI analyses which display attractive host-guest interactions in both the polar and non-polar regions present in **7** (**Supplementary Figure S37**).\

a **b**

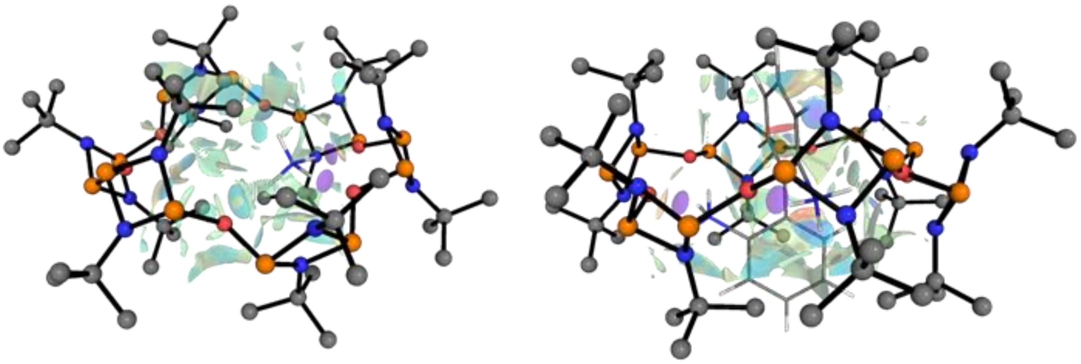

Supplementary Figure S37: Noncovalent interaction computed at ω-B91xD/6-31G(d,p) level for for NH_4^+ (top) and [1,1'-biphenyl]-2,2'-diaminium (bottom) host-guest adduct (attractive interactions are shown in purple).

In order to further rationalize the calculated host properties of these species, Electrostatic surface potential (ESP) analysis of **18C6**, **21C7** and **7** were carried out. In the case of **18C6** and **21C7**, the highest electron density is concentrated in the internal cavity of the crow ethers (**Supplementary Figures S38** and **S39**). Additionally, the electron-poor regions of the crown ethers are located in the outer region within the plane defined by the bridging carbon atoms. A more detailed analysis of the surface shows that all the minima are indeed localized in the plane defined by all the oxygen atoms (**Supplementary Figures S38-S39**), while the maxima of the surface are located near the outer bridging CH_2 atoms. This highly localized charge distribution is in good agreement with the capacity of both crow ethers to stabilize only positively charged species in the internal region.

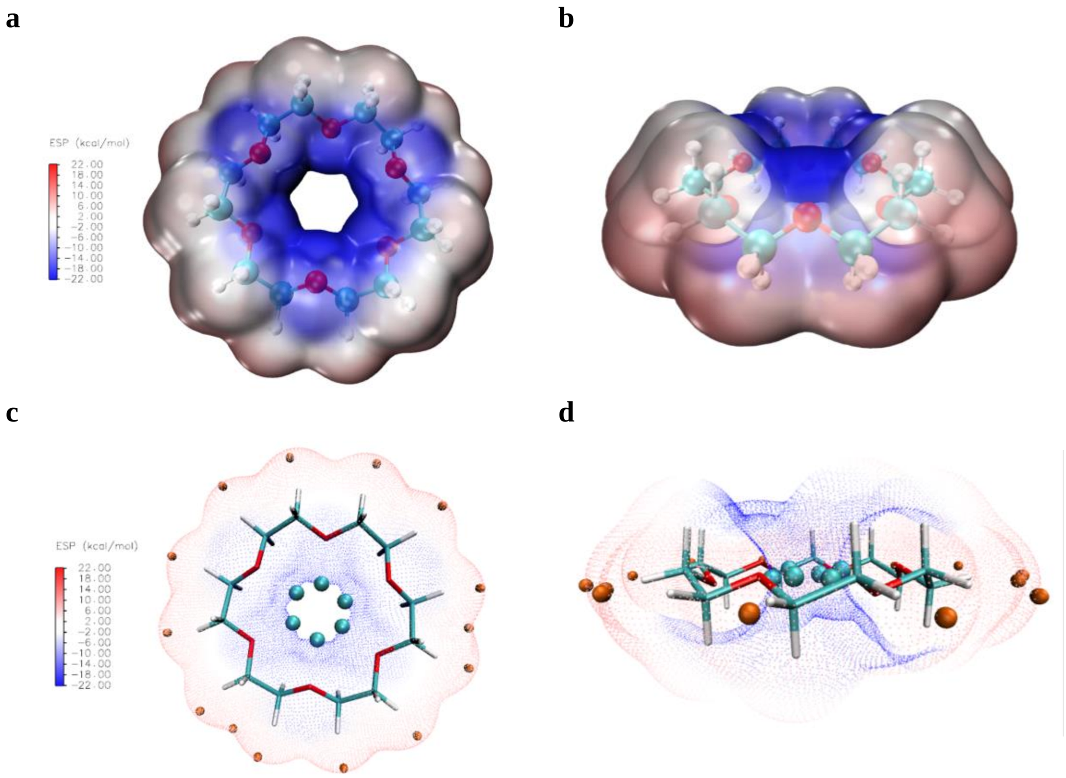

Supplementary Figure S38. ESP maps for 18C6 (Isovalue = 0.01) (**a** and **b**, top and side views, respectively) and ESP maps for **18C6** including maxima and minima (**c** and **d**, top and side views, respectively)

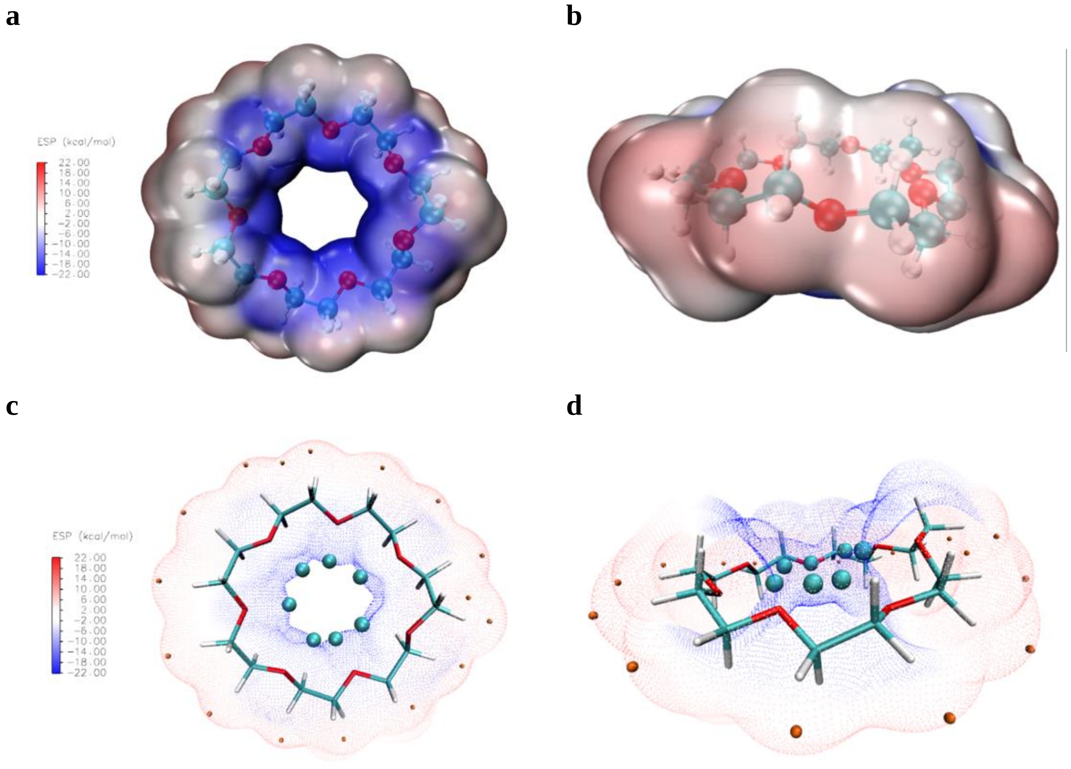

Supplementary Figure S39. ESP maps for **21C7** (Isovalue = 0.01) (**a** and **b**, top and side views, respectively) and ESP maps including for **18C6** including maxima and minima (**c** and **d**, top and side views, respectively)

In the case of **7**, the ESP map reveals a highly negative region in the internal cavity of the host, compatible with the favourable interaction with positively charged species in this region (**Supplementary Figure S40**). In contrast with the observations made in the case of the crown ethers, the analysis of the maxima and minima of the ESP reveals a much less localized of the minima (**Supplementary Figure S40)**, probably due to both the polarization of the ^{t}Bu groups and the presence of electronegative nitrogen atoms above an under the plane defined by the oxygen atoms. This uneven distribution of ESP minima is in good agreement with the higher stabilization of [1,1'-biphenyl]-2,2'-diaminium with **7** compared with both crown ethers, in which the stabilization of positively charged species is only possible in the internal plane defined by the oxygen atoms. Additionally, 7 also shows a positively charged region in the top and bottom regions of the plane defined by the oxygen atoms, in good accord with the amphiphilic character observed in the stabilization of neutral [1,1'-biphenyl]-2,2'-diamine, in which the relatively electron poor aryl groups can be stabilized by these upper and lower positive regions.

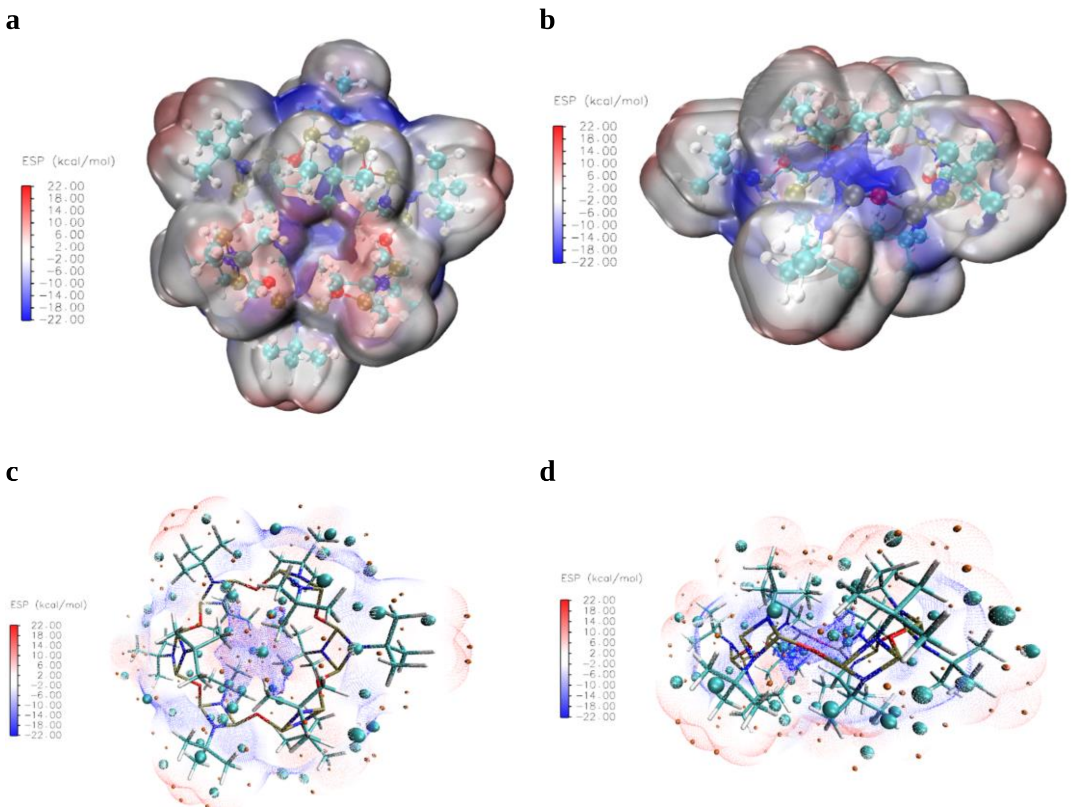

Supplementary Figure S40. Top view (**a**) and side view (**b**) of ESP maps for 7 (Isovalue = 0.01) and ESP maps including maxima and minima (Isovalue = 0.01) top (**c**) and side (**d**) views

4 Supplementary References

[1] Jefferson, R.; Nixon, J. F.; Painter, T. M.; Keat, R.; Stobbs, L. *J. Chem. Soc., Dalton Trans.*, **1973**, 1414.

[2] Scherer, O. J.; Andres, K.; Krüger, C.; Tsay, Y.-H.; Wolmerhäser, G. *Angew. Chem. Int. Ed.*, **1980**, *19*, 571.

[3] SMART version 5.628; Bruker AXS Inc., Madison, WI, USA, 2001.

[4] Sheldrick, G. M. SADABS V2014/4 (Bruker AXS Inc.) University of Göttingen, Göttingen, Germany, 2014.

[5] SHELXL-2014/6 (Sheldrick, 2014) ; Bruker AXS Inc., Madison, WI, USA, 2014.

[6] Becke, A. D. Density-functional thermochemistry. V. Systematic optimization of exchange correlation functionals. *J. Chem. Phys*. **1997**, *107*, 8554–8560.

[7] Chai, J.-D.; Head-Gordon, M. Long-range corrected hybrid density functionals with damped atom–atom dispersion corrections. *Phys. Chem. Chem. Phys*. **2008**, *10*, 6615–6620.

[8] Gaussian 16, Revision B.01, Frisch, M. J.; Trucks, G. W.; Schlegel, H. B.; Scuseria, G. E.; Robb, M. A.; Cheeseman, J. R.; Scalmani, G.; Barone, V.; Petersson, G. A.; Nakatsuji, H.; Li, X.; Caricato, M.; Marenich, A. V.; Bloino, J.; Janesko, B. G.; Gomperts, R.; Mennucci, B.; Hratchian, H. P.; Ortiz, J. V.; Izmaylov, A. F.; Sonnenberg, J. L.; Williams-Young, D.; Ding, F.; Lipparini, F.; Egidi, F.; Goings, J.; Peng, B.; Petrone, A.; Henderson, T.; Ranasinghe, D.; Zakrzewski, V. G.; Gao, J.; Rega, N.; Zheng, G.; Liang, W.; Hada, M.; Ehara, M.; Toyota, K.; Fukuda, R.; Hasegawa, J.; Ishida, M.; Nakajima, T.; Honda, Y.; Kitao, O.; Nakai, H.; Vreven, T.; Throssell, K.; Montgomery, J. A., Jr.; Peralta, J. E.; Ogliaro, F.; Bearpark, M. J.; Heyd, J. J.; Brothers, E. N.; Kudin, K. N.; Staroverov, V. N.; Keith, T. A.; Kobayashi, R.; Normand, J.; Raghavachari, K.; Rendell, A. P.; Burant, J. C.; Iyengar, S. S.; Tomasi, J.; Cossi, M.; Millam, J. M.; Klene, M.; Adamo, C.; Cammi, R.; Ochterski, J. W.; Martin, R. L.; Morokuma, K.; Farkas, O.; Foresman, J. B.; Fox, D. J. Gaussian, Inc., Wallingford CT, 2016.
