# Supplementary material for: Size-control in the synthesis of oxo-bridged phosphazane macrocycles via a modular addition approach
Source: Commun Chem. 2021 Feb 22;4:21. doi: 10.1038/s42004-021-00455-9 (PMC9814222; doi:10.1038/s42004-021-00455-9)
Supplement: Supplementary file 2 — Description of Additional Supplementary Files [file 42004_2021_455_MOESM2_ESM.pdf]

Description of Additional Supplementary Files

File Name: Supplementary Data 1
Description: Crystallographic information file for compound 4

File Name: Supplementary Data 2
Description: Crystallographic information file for compound 5

File Name: Supplementary Data 3
Description: Crystallographic information file for compound 7
